# Antitumor activity of rucaparib plus PLX038A in serous endometrial carcinoma

**DOI:** 10.1186/s13046-025-03406-7

**Published:** 2025-05-19

**Authors:** Xiaonan Hou, Valentina Zanfagnin, Conway Xu, Erik Jessen, Yuanhang Liu, Chen Wang, Yajue Huang, Shaun D. Fontaine, Daniel V. Santi, Gerardo Colon-Otero, Sarah E. Gill, Gretchen E. Glaser, Kristina A. Butler, Jamie N. Bakkum-Gamez, Sean C. Dowdy, Ann L. Oberg, Melissa C. Larson, Hunter J. Atkinson, Laura N. Duffield, Kevin L. Peterson, Scott H. Kaufmann, S. John Weroha

**Affiliations:** 1 Division of Medical Oncology, Mayo Clinic, Rochester, MN 55905 USA; 2 Department of Health Sciences Research, Mayo Clinic, Rochester, MN 55905 USA; 3 Division of Anatomic Pathology, Mayo Clinic, Rochester, MN USA; 4 ProLynx LLC, 455 Mission Bay Blvd, South San Francisco, CA 94158 USA; 5 Hematology/Oncology, Mayo Clinic Florida, Jacksonville, FL 32224 USA; 6 Department of Obstetrics and Gynecology, Mayo Clinic, Rochester, MN 55905 USA; 7 Division of Gynecologic Oncology, Department of Obstetrics and Gynecology, Mayo Clinic, MN, 55905 USA; 8 Medical & Surgical Gynecology Department, Mayo Clinic, Phoenix, AZ USA; 9 Department of Quantitative Health Sciences, Division of Computational Biology, Mayo Clinic, Rochester, MN 55905 USA; 10 Department of Quantitative Health Sciences, Division of Clinical Trials and Biostatistics, Mayo Clinic, Rochester, MN 55905 USA; 11 Division of Oncology Research, Mayo Clinic, Rochester, MN 55905 USA; 12 Department of Molecular Pharmacology & Experimental Therapeutics, Mayo Clinic, Rochester, MN 55905 USA; 13 Department of Oncology, Mayo Clinic College of Medicine, 200 First St. SW, Rochester, MN 55905 USA

**Keywords:** Endometrial cancer, Xenografts, PARP inhibitor, Rucaparib, Homologous recombination, DNA repair, SN-38

## Abstract

**Background:**

Serous endometrial cancer (SEC) is a genomically and morphologically distinct endometrial cancer (EC) subtype with a poor progression-free and overall survival. The development of novel therapies is needed to improve outcomes.

**Methods:**

We used serous and serous-like EC patient-derived xenografts (PDXs) to test a novel drug combination in vitro and in vivo: rucaparib and pegylated SN-38 (PLX038A). Sensitivity to treatment was correlated with indicators of homologous recombination (HR) deficiency. Efficacy in fresh primary patient tumors was also tested ex vivo.

**Results:**

Five of eight PDXs had genomic instability scores ≥ 42, but only one of these five had evidence of HR deficiency in assays of irradiation-induced RAD51 foci formation. Moreover, PARP inhibitor (PARPi) monotherapy failed to induce regressions in any of the five SEC models treated with rucaparib in vivo, suggesting limited clinical activity of PARPi in SEC. In further studies, we assessed the response of these models to the sustained release topoisomerase 1 inhibitor, PLX038A, as monotherapy and in combination with rucaparib ex vivo and in vivo. Results of these studies showed that PLX038A had limited monotherapy activity, but combination therapy induced significant regressions in two of five SEC PDXs and markedly slowed tumor growth in the other three regardless of underlying homologous recombination repair deficiency. In addition, 11 of 20 (55%) primary tumors showed synergy with rucaparib + SN-38.

**Conclusions:**

Collectively, these studies identify a set of genomically characterized PDX models for preclinical testing of potential SEC therapies and a therapeutic combination that warrants further preclinical investigation.

**Supplementary Information:**

The online version contains supplementary material available at 10.1186/s13046-025-03406-7.

## Background

Endometrial cancer (EC) is the most common gynecological malignancy in the USA [1]. Although most patients with early-stage disease are cured with surgery ± radiation therapy, recurrences are common for patients with advanced stage or aggressive histologic subtypes such as serous carcinomas [2–4]. Because standard chemotherapy regimens have limited efficacy in serous EC (SEC), this disease is associated with a high risk of recurrence, regardless of stage [5]. At present, therapeutic options for recurrent SEC are also limited. Pembrolizumab monotherapy has been FDA-approved for tumors with microsatellite instability, but most SECs are microsatellite stable [6, 7]. With lenvatinib plus pembrolizumab, which is FDA-approved for recurrent microsatellite stable EC [8], the objective response rate (ORR) is 40% (14/35) for SEC, but most patients experience grade 3 + adverse events at the recommended lenvatinib starting dose of 20 mg daily [8]. Another targeted agent, trastuzumab, prolongs progression-free survival in HER2-positive SECs [9], but the largest study of SECs (*n* = 2159) reported only a 17% incidence of *HER2* amplification [7]. Accordingly, SECs are still in need of improved therapies.

SECs exhibit several similarities to high grade serous ovarian cancer (HGSOC). SECs not only have a high prevalence of *TP53* alterations, but also occur more commonly in *BRCA1/2* mutation carriers than in the general population [10]. Moreover, germline alterations in other homologous recombination (HR) genes such as *RAD51C/D*,* BRIP1*,* BARD1*,* CHEK1*,* ATM*, and *NBN* also occur in EC patients [11]. In aggregate, somatic HR gene mutations have also been reported in 34% of ECs [12], suggesting that a subset of ECs is HR-deficient. In a separate study, the Cancer Genome Atlas has defined a molecular subtype of serous-like ECs based on molecular characteristics rather than histologic features [6]. Importantly, 15% of these serous-like ECs harbor a mutational signature associated with defective HR [13]. HR deficiency has also been demonstrated as inability to form RAD51 foci ex vivo in six of six (100%) SECs and carcinosarcomas assayed [14]. Moreover, a retrospective study has identified loss of heterozygosity (LOH) as a potential marker of HR deficiency in 22% of 2,159 SECs examined [7]. Collectively, these results indicate that a subset of serous-like ECs share molecular characteristics with HGSOC, raising the possibility that therapeutic approaches in ovarian cancer might also be effective in serous-like ECs.

Studies have demonstrated over the past decade that poly(ADP-ribose) polymerase inhibitors (PARPis) are active when administered as monotherapy or maintenance therapy to patients with HGSOC, with the greatest efficacy observed in cancers harboring *BRCA1/2* mutations or other HR defects. Moreover, PARPis are more active in the frontline [15–17] rather than recurrent setting [18–21]. The similarity of SECs to HGSOCs raises the possibility that PARPis might represent effective treatment for HR-deficient ECs as well.

Several observations also provide the rationale for combining PARPis with topoisomerase 1 (TOP1) inhibitors (TOP1is) in EC. First, TOP1is have activity in EC, with ORRs of 20% for topotecan [22], 36% for irinotecan [23], and 57% for the TOP1i-containing antibody-drug conjugate trastuzumab deruxtecan specifically in HER2 + EC [24]. Second, multiple PARPi/TOP1i combinations have demonstrated synergy in other cancers [25–30], reflecting both inhibited repair of TOP1i-induced damage as a consequence of PARP trapping [29] and diminished recruitment of the phosphodiesterase TDP1 (Tyrosyl-DNA phosphodiesterase 1) that contributes to reversal of TOP1-DNA covalent complexes [31].

The goals of the present study were to develop and characterize EC patient-derived xenograft (PDX) models, determine the efficacy of the PARPi rucaparib in a spectrum of HR proficient and deficient PDXs, and investigate the antineoplastic activity TOP1i/rucaparib combination. These studies not only showed that HR deficiency occurs in a subset of EC PDXs, but also demonstrated synergy of a rucaparib/TOP1i combination in SEC cell lines in vitro, primary patient SECs cultured ex vivo, and SEC PDXs in vivo.

## Methods

### Materials

SN-38 for in vitro and ex vivo studies was purchased from Bio-Techne (Minneapolis, MN). Rucaparib for i*n vitro* and ex vivo studies was purchased from Chemietek (Indianapolis, IN). Rucaparib for in vivo animal study was donated by patients from unneeded, non-expired clinical supplies. PEGylated SN-38 (PLX038A), synthesized as previously described [32], was formulated as a solution in isotonic acetate buffer (pH 5.0; 143 mM NaCl, 20 mM NaOAc) and contained 1.22 mM SN-38 equivalents (0.305 mM conjugate; 4 equivalent SN-38 molecules/conjugate).

### Establishment of EC PDXs

Under the aegis of protocols approved by the Mayo Clinic Institutional Review Board (IRB), all patients gave written consent to participate prior to primary surgery or clinical biopsy for recurrent disease (#09-008768, #15-007262, or #17-007946). IRB approvals are in accordance with the U.S. Department of Health and Human Services federal policy for the Protection of Human Subjects (the Common Rule), published in the Federal Register on January 19, 2017. Tumors were minced in McCoy’s 5 A medium, supplemented with penicillin/streptomycin and rituximab (10 mg/kg) (Rituxan; Genentech, Inc., San Francisco, CA) to prevent unintentional lymphoproliferative tumors [33], and injected intraperitoneally [34] into female SCID-bg mice (C.B.-17/IcrHsd-Prkdc^scid^ Lyst^bg^; ENVIGO), following procedures that were approved by the Mayo Clinic Animal Care and Use Committee in facilities that are accredited by the American Association of Laboratory Animal Care. Mice were monitored weekly for tumor engraftment and euthanized when moribund criteria were met. Minced tumors were cryopreserved for subsequent studies as a 1:1 suspension in freezing medium (39% FBS, 10% dimethyl sulfoxide, 1% penicillin/streptomycin in McCoy’s 5 A medium). Key clinical characteristics of the 10 PDX tumors with functional assessment of homologous recombination (HR) activity are described in Supplemental Table 1.

### Derivation of genomic instability score and variant calling

Detailed methods are provided in Supplemental Methods. Briefly, the analysis of whole genome sequencing (WGS) data from PDX tumors was conducted using the Mayo Bioinformatics in-house pipeline named GenomeGPS (GGPS), a comprehensive toolset for the alignment and analysis of DNA sequencing data. Reads from murine DNA were removed prior to GGPS processing by aligning all reads to either the human (hg38) or mouse (mm10) genomes. All subsequent analyses were performed on human-specific and ambiguous reads, but mouse-specific and completely-conserved reads were omitted. ***For variant calling***, HaplotypeCaller was used with a minimum allele frequency of 0.2. Somatic variant calling was conducted on the realigned BAM files using GATK Mutect2 in tumor-only mode, with GNOMAD employed as the reference database [35]. The ***genomic instability score (GIS)*** was computed in four steps. *First*, copy number variation (CNV) quantification and segmentation was performed using Wandy to assess for deviations from the median coverage for 10 kb genome bins. The bin-level normalized coverage was converted to a log fold change based on the median normalized coverage for each sample. *Second*, regions of allelic imbalance were identified by detecting deviations in the absolute change of alternative allele concentration (AAC) of all heterozygous SNPs. SNPs with allele concentrations between 0.05 and 0.95 were classified as non-homozygous, and the average absolute deviation of AAC values from the median value was calculated as a log fold change relative to the median. *Third*, allele-specific CNV calls were based on a combined AAC segmentation and CNV segmentation while accounting for tumor purity, balanced deletions, and duplications/amplifications. *Fourth*, ScarHRD [36] was adapted for WGS allele-specific CNV calls to count the tumor number of events meeting criteria for loss of heterozygosity (LOH), large scale transitions (LST), telomeric allele imbalance (TAI).

### RAD51 foci

Single cell PDX suspensions were created from fresh PDX tissue using the gentleMACS™ Dissociator (130-093-235, Miltenyi Biotec, Germany). As negative and positive RAD51 staining controls, PEO1 (HR deficient, *BRCA2* mutation) and PEO4 (HR proficient, *BRCA2* revertant) cells were cultured in DMEM containing 10% (vol/vol) heat-inactivated fetal bovine serum (FBS), 100 µM nonessential amino acids. Cells were allowed to adhere to coverslips overnight before exposure to irradiation (10 Gy) from a ^137^Cs source. After a 6 h incubation, cells were washed twice with cold PBS, fixed in cold 4% (wt/vol) paraformaldehyde for 15 min, permeabilized with 0.5% (wt/vol) Triton X-100 in PBS, and blocked overnight with 5% (v/v) goat serum in PBS. Coverslips were incubated with rabbit monoclonal anti-RAD51 (Abcam ab133534, 1:4000) and mouse monoclonal anti-geminin (Abcam ab104306, 1:100) in blocking buffer overnight at 4 °C, washed 6 times over 20 min with wash buffer (PBS, 0.1% Triton X-100 and 0.1% bovine serum albumin), incubated with Alexa fluor 488 goat anti-rabbit IgG (Invitrogen A11008, 1:1000) and Alexa fluor 568 goat anti-mouse IgG (Invitrogen A11004, 1:1000) in blocking buffer for 1 h at 21 °C in the dark, washed, and mounted in VECTASHIELD^®^ antifade medium with DAPI (Vector Labs CA, 94010). Samples were examined on a Zeiss Axiovert microscope with a N.A. 1.40 100× lens and photographed on a Zeiss Axiocam MRm CCD camera using Zeiss Zen software. RAD51 foci were quantified manually in a blinded fashion in at least 100 geminin-positive cells per slide and considered positive if ≥ 10 RAD51 foci were visible [37]. Alternatively, adherent PDX cultures treated with rucaparib (25 µM), SN-38 (0.5 µM) or the combination for 24 h were incubated with anti-RAD51 and rabbit monoclonal anti phospho-Ser139-histone H2A.X (Cell Signaling Technology 9718 S, 1:400) followed by visualization as described above.

### Cell culture, clonogenic assays and flow cytometry

ARK-1, ARK-2, SPAC1-L, and SPAC1-S SEC cell lines [38, 39] from Gottfied Konecny (University of California Los Angeles) cultured in Dulbecco’s Modified Eagle Medium supplemented with 10% heat-inactivated FBS, 100 U/ml penicillin and 100 µg/ml streptomycin (medium A) were seeded at 750 (ARK-2 cells), 1000 (ARK-1 or SPAC1-L cells) or 1500 cells (SPAC1-S cells) per dish in triplicate 60 mm dishes containing 3 ml medium A. Eighteen hours later, drugs were added at the indicated concentrations. Colonies (defined as > 50 cells) were allowed to form for 6–9 days in the continuous presence of drug, stained with Coomassie blue and manually counted. All assays were performed at least three times independently, and graphed results indicate the mean ± standard deviation of means from the individual experiments.

To assay for apoptosis, 70,000 cells were seeded in 10 ml medium A in 100 mm dishes, allowed adhere overnight, exposed to various drug concentrations for 4 days, trypsinized, stained with 50 µg/ml propidium iodine in 0.1% (w/v) sodium citrate, and subjected to flow microfluorimetry as described by Nicoletti et al. [40]. Apoptotic cells were detected as subdiploid events. Alternatively, cells were stained with Annexin V and detected as previously described [40].

### Ex vivo tumor 3D culture

Fresh EC PDXs were dissociated with the gentleMACS™ Dissociator and plated in ultra-low attachment 384 well microplates (CLS3571, Corning Life Science, USA) in DMEM supplemented with 15% FBS. After 24 h, SN-38 (0.5, 0.25, 0.125, 0.0625 or 0.03125 µM), rucaparib (25, 12.5, 6.25, 3.125, or 1.56 µM), or the combination (at the same concentrations) were added to triplicate wells and in three separate experiments. After 72 h, response was determined by the RealTime-Glo MT Cell Viability Assay (G9711, Promega Corporation, USA) in GloMax Discover System (GM3000, Promega Corporation, USA).

Fresh primary patient hysterectomy specimens from consecutive patients with newly diagnosed EC were provided under an approved Mayo IRB protocol (#17-007946) following the same ethics standards for creation of PDX models. Because molecular classification was not available at the time of tumor collection, eligibility criteria were limited to patients with serous or presumed serous-like histology (carcinosarcoma, high-grade endometrioid). Tissues were processed and treated as above for ex vivo PDX 3D culture. Combination index calculations were calculated as below.

### Combination index calculations

Combination indices (CIs) [41] were calculated using CalcuSyn software, v2.1 (Biosoft, Cambridge, UK) under the assumption that effects are mutually exclusive, which yields results comparable to isobologram analysis [42]. CI > 1 indicates antagonism, CI = 1 indicates additivity, and CI < 1 indicates synergy. For clonogenic assays and RealTime-Glo assays, fraction affected indicates mean decrease in signal compared to the control. For flow cytometry, fraction affected represents the percentage of subdiploid cells detected out of 30,000 events collected by flow microfluorimetry.

### PDX efficacy studies

Cryogenically preserved PDXs were reestablished in female SCID Beige mice as previously described [34]. Briefly, 0.1–0.2 cc of minced tumor in 1:1 ratio with McCoy’s 5 A Modified Medium (MT-10-050-CV, Corning Life Science, USA) was injected intraperitoneally. When tumor cross-sectional area by transabdominal ultrasound reached 0.3–0.5 cm^2^, mice were randomized to saline control, rucaparib (150 mg/kg daily gavage), PLX038A (15 µmol/kg IP every 2 weeks), or combined rucaparib and PLX038A for eight weeks [34, 37, 43]. A dose titration of PLX038A was previously performed in breast cancer cell line xenografts and revealed that 4 µmol/kg had anti-tumor activity while 120 µmol/kg resulted in near-complete resolution of tumors [44]. Ultimately, PLX038A at 15 µmol/kg was chosen in collaboration with ProLynx to maximize the potential to observe a difference between combination therapy and monotherapy. Tumor size by ultrasound was measured weekly and plotted as the mean tumor area per cohort relative to the mean starting size of the cohort. Statistical analysis of PDX efficacy studies performed as previously described [45] is described in detail in the Supplementary methods.

### TP53 immunohistochemistry (IHC)

Primary patient tumor specimens from ten PDX studied were stained for p53 by the Pathology Research Core (Mayo Clinic, Rochester, MN) using a Leica Bond RX stainer (Leica). Formalin fixed, paraffin-embedded tissues were sectioned at 5 microns, mounted on charged slides, and dried overnight. Slides stained for p53 were retrieved for 20 min using Epitope Retrieval 1 (Citrate; Leica) and incubated in Protein Block (Dako) for five minutes. The monoclonal primary antibody p53 (Clone DO-7; Dako), which can recognize both wild type and mutant forms of human p53 protein, was diluted at 1:2000 and incubated for 15 min. Immunostaining was visualized by incubating slides for 10 min in DAB and DAB buffer (1:19 mixture) from the Bond Polymer Refine Detection System (Leica). Slides were counterstained for five minutes using a 1:1 mixture of Schmidt hematoxylin (Mayo Department of Laboratory Medicine Preparation and Processing Laboratory) and molecular biology grade water, removed from the stainer, rinsed in tap water for 3 min, dehydrated in increasing concentrations of ethanol, cleared in 3 changes of xylene, and permanently covered in xylene-based medium. Interpretation was performed by a clinical gynecologic oncology pathologist. An aberrant pattern was defined as strong uniform staining or an absence of staining in epithelial carcinoma cells. A wildtype pattern was heterogenous staining in epithelial cells.

## Results

### Derivation of EC PDXs and assessment of genomic instability score (GIS)

To assess potential therapies for EC patients, preclinical models were needed. Accordingly, 30 intraperitoneal EC PDXs were generated from 51 uterine cancers (adenocarcinoma and sarcoma) and the engraftment rate was 58.8% (Supplemental Fig. 1). Of the tumors that engrafted, 9 were derived from imaging-guided biopsies of relapsed EC and 21 from tumors collected at the time of primary surgery. The distribution of histology among these PDXs included endometrioid (*n* = 9 grade 1 or 2, *n* = 6 high grade), serous (*n* = 8), clear cell (*n* = 2), mixed histology (*n* = 3) (one serous and clear cell, one mucinous and endometrioid, one serous and endometrioid), and carcinosarcoma (*n* = 2). For all engrafted PDXs, the median time to initial engraftment (defined by the first mouse of each PDX line to become moribund with tumor) was 7.5 months after transplantation from the patient. After the first passage, the subsequent engraftment rate was 100% and the time to moribund was shorter, indicating an increased growth rate. The most common site of engraftment was in the pelvis as a single solid mass, but attachment to other abdominal sites was also observed: bowel, mesentery, visceral pleura of liver, spleen, diaphragm, and omentum. Tumor-associated morbidity was observed in one PDX line that had a tendency to encase the bowel or mesentery causing bowel dysfunction, which is a known clinical problem for patients with recurrent EC. Another potential cancer-related morbidity is ascites, which can be seen in one-third of patients with peritoneal spread [46]. In our EC PDX models, ascites in ≥ 1 animal was observed in 27% of PDX lines.

**Fig. 1:**
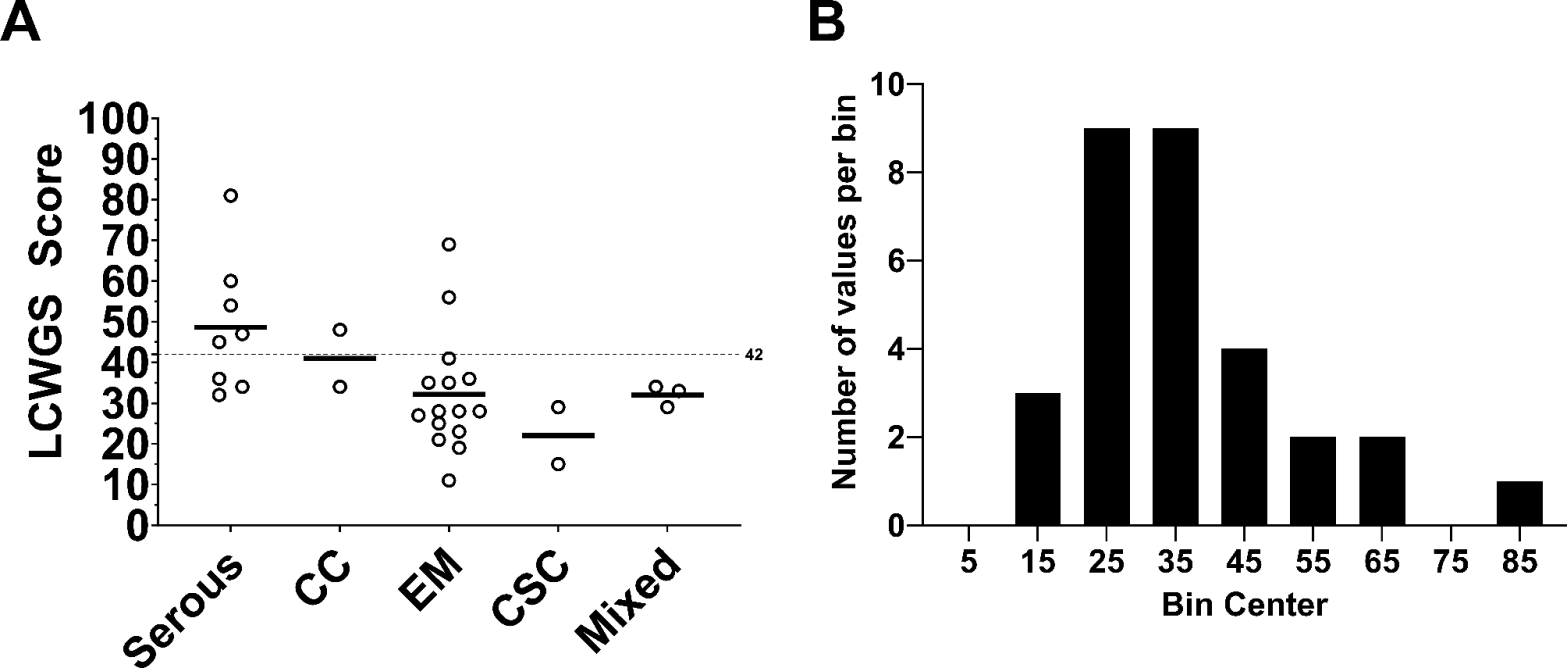
Low-coverage whole genome sequencing (LCWGS) genomic instability score (GIS). **A**) Thirty EC PDXs with serous, clear cell (CC), endometrioid (EM), carcinosarcoma (CSC), or mixed histology are shown. **B**) GIS distribution across PDXs

Based on previous studies, a subset of ECs was expected to exhibit molecular indicators of HR deficiency, also known as a *genomic instability score* (GIS), but commercial assays have not been evaluated in EC [47]. Using established methods for copy number and genomic instability analysis based on low-coverage whole genome sequencing (LCWGS) [48, 49], a GIS was derived from a combined assessment of telomeric allelic imbalance, large state transitions, and loss of heterozygosity [47]. Although a GIS threshold value has not yet been defined for EC, 8 of 30 (26.6%) EC PDX tumors had a GIS ≥ 42, which is the threshold for defining ovarian cancers as GIS-positive. The percentage of EC PDXs with a GIS ≥ 42 was highest in serous (5 of 8) followed by clear cell (1 of 2), endometrioid (2 of 15), mixed histology (0 of 3) and carcinosarcoma (0 of 2) (Fig. 1A). The GIS distribution among these histologically diverse tumors ranged from 0 to 81. Due to the small sample size, a bimodal distribution of scores was not observed (Fig. 1B), as reported for the Myriad HRD assay with a larger sample size [47, 50].

**Fig. 2:**
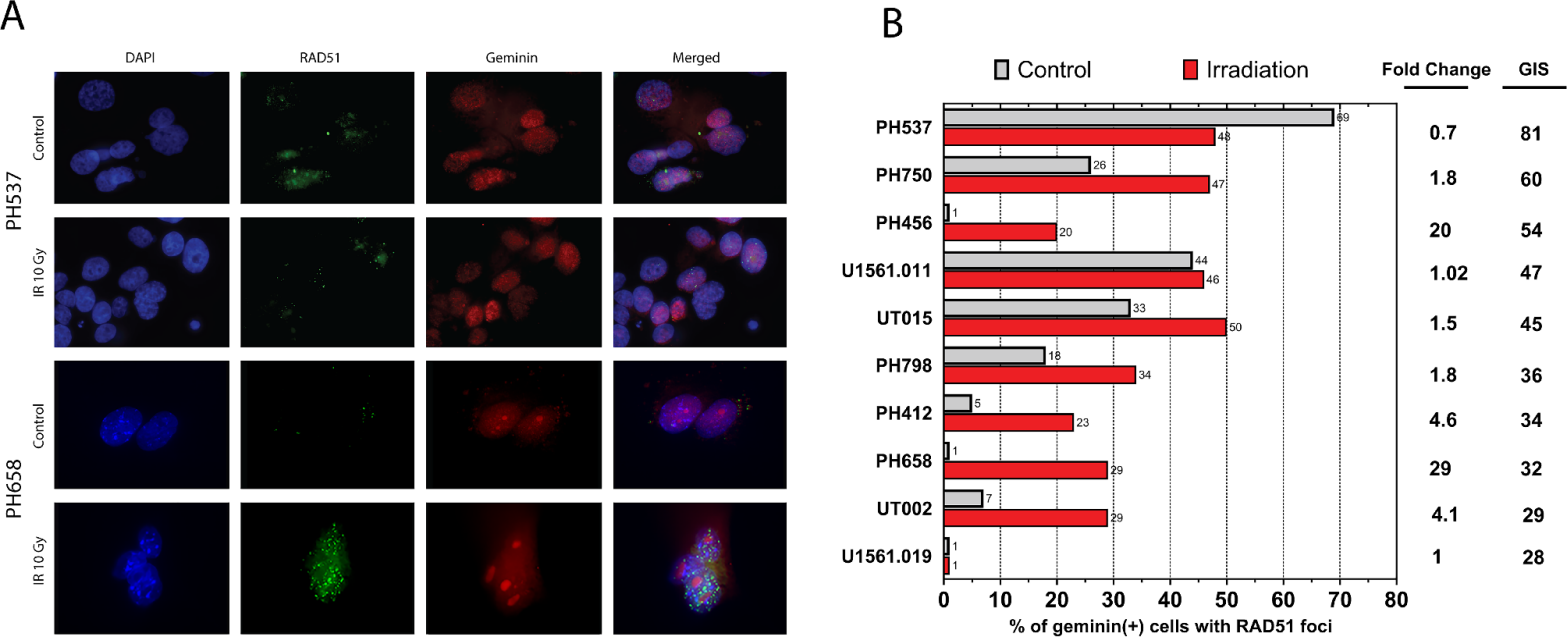
Functional assessment of homologous recombination (HR) activity in serous and serous-like EC PDX tumors. **A**) Representative immunofluorescence staining of RAD51 foci in EC PDX models with a high (PH537) or low (PH658) genomic instability score (GIS). **B**) Percentage of cells with RAD51 foci after radiation compared to un-irradiated controls. GIS and fold change in foci percent (irradiated/control) are shown

HR pathway function was assessed by a RAD51 foci formation assay [37]. PEO1 cells (*BRCA2*-mutant) and PEO4 cells (*BRCA2* revertant [51]) served as negative and positive controls, respectively. Analysis focused on a subset of 10 ECs (eight serous, one carcinosarcoma, one high grade endometrioid) to determine if they have evidence of HR pathway inactivation similar to serous ovarian cancers. After PDXs were resected and dissociated to establish a short-term 2D culture, DNA double-strand breaks were induced with ionizing radiation and the formation of RAD51 foci was used as an indicator of intact HR DNA repair. Geminin was used to identify cells in S and G2 phases, when HR repair can occur. Because non-irradiated cells exhibited foci at baseline, ECs were only considered HR-deficient if radiation failed to induce additional foci beyond baseline. As expected, PH537, which had the highest LCWGS-GIS (suggesting HR deficiency), did not exhibit an irradiation-induced increase in RAD51 foci (Fig. 2A). Conversely, PH658 had a low LCWGS-GIS and showed a radiation-induced increase in RAD51, consistent with HR proficiency. Collectively 4 of 5 PDXs with a GIS score < 42 formed radiation-induced RAD51 foci. Notably, however, 3 of 5 PDXs with a GIS score ≥ 42 also exhibited a radiation-induced increase in RAD51 foci. For example, PH750 (second highest GIS) formed more RAD51 foci with radiation, while U1561.019 (lowest GIS) lacked radiation-induced RAD51 foci (Fig. 2B), again indicating that high GIS score and lack of RAD51 foci do not consistently track together. PEO1 and PEO4 cell lines, derived from a single ovarian cancer patient, were used as negative and positive controls for formation of RAD51 foci (Supplemental Fig. R2).

To confirm each of the 10 included PDX tumors were derived from patients with histologically and/or molecularly serous/serous-like cancers, p53 protein expression was stained by IHC and evaluated as recommended in clinical practice [52]. Primary patient samples were used except for U1561.011, where PDX tissue was stained because the source tumor was unavailable. An aberrant p53 pattern was characterized by a complete absence of staining (PH412 and U1561.019) or strong diffuse staining specifically in the epithelial carcinoma cells (PH456, PH537, PH750, PH798, UT002, UT015, U1561.011). A wildtype pattern of heterogenous staining in epithelial carcinoma cells was observed in PH658, despite the serous histology (Supplemental Fig. 3). Paired hematoxylin and eosin (H&E) stain showed elongated and irregular glands with slit-like luminal spaces (PH412, UT015 and UT002) or more rounded glands with smooth luminal borders and a solid growth pattern (PH658). The cells display marked nuclear pleomorphism, macronucleoli, and conspicuous mitotic activity (Supplemental Fig. 4). In addition, the patterns observed in the primary patient tumor were recapitulated in the PDX tumor.

### Rucaparib synergizes with SN-38 in SEC cell lines

**Fig. 3:**
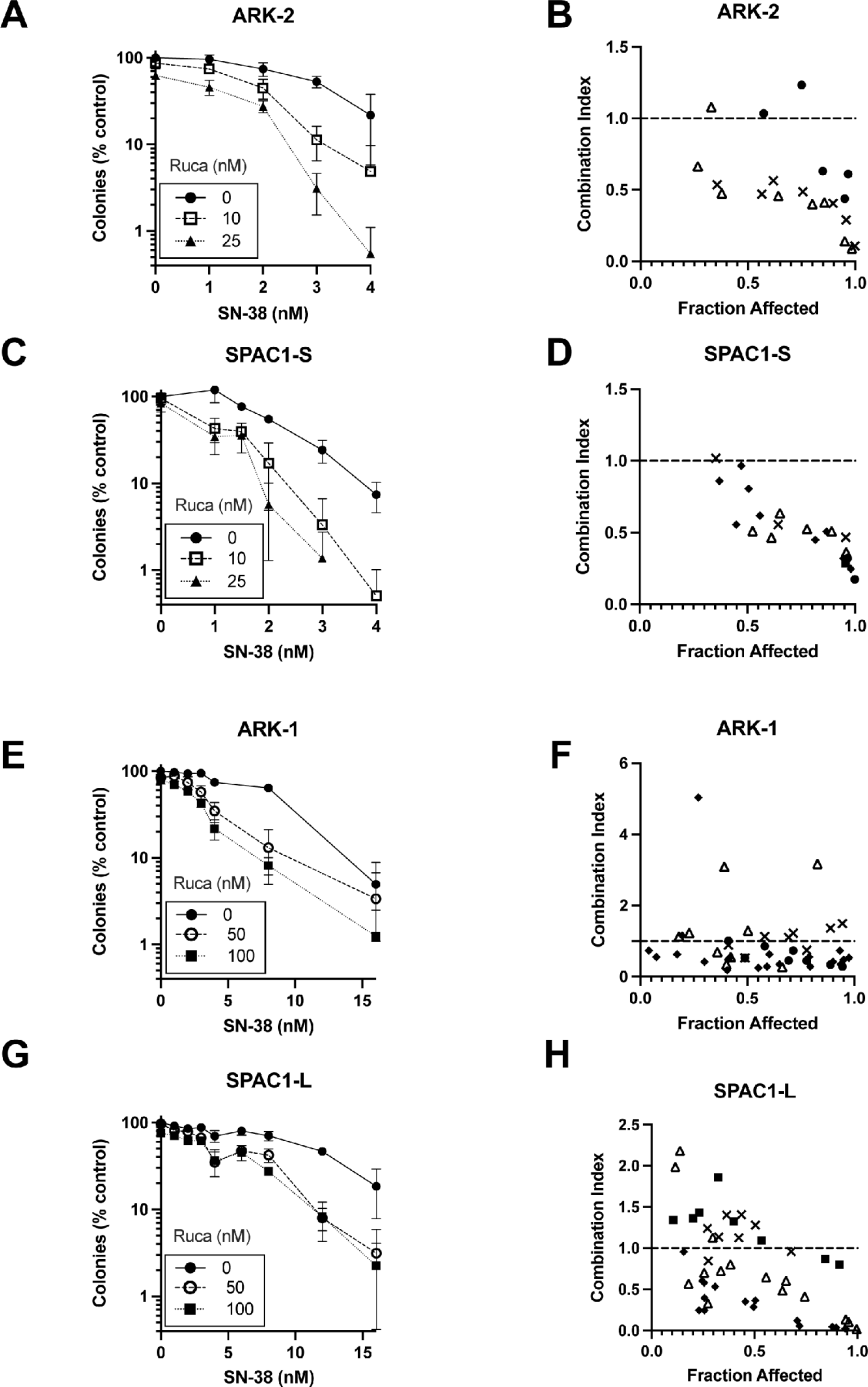
Clonogenic survival of SEC cell lines in different concentrations of SN-38 and rucaparib. **A**), **C**), **E**), **G**), cells were cultured with continuously in the indicated drug concentrations for 6–9 days, stained and counted. Error bars, mean $\:\pm\:$ SEM of five independent experiments. **B**), **D**), **F**), **H**), combination index (CI) for the SN-38 + rucaparib drug combination in SEC cell lines. Different shapes indicate results from each of 3–5 independent experiments

**Fig. 4:**
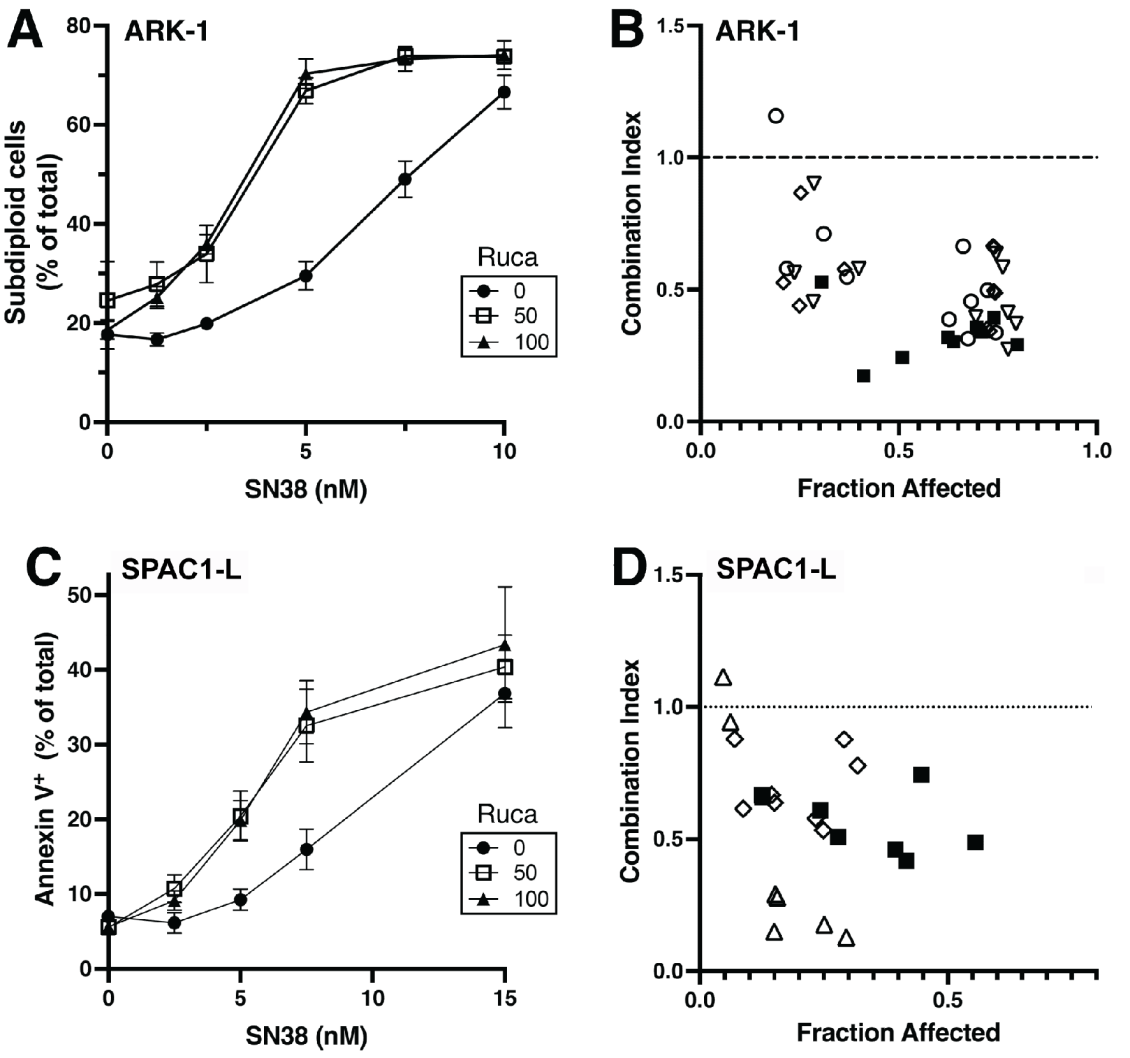
Induction of apoptosis in SEC cell lines at different concentrations of SN-38 and rucaparib. **A**) and **C**), cells were cultured with continuous exposure to the indicated drug concentrations for 4 days, stained with propidium iodide in sodium citrate (**A**) or Annexin V (**C**), and subjected to flow microfluorimetry. Error bars indicate mean $\:\pm\:$ SEM of 3–4 independent experiments. **B**) and **D**), combination index (CI) for the SN-38 + rucaparib drug combination. Different shapes indicate results from each of 3–4 independent experiments

Given the inconsistent evidence for functional HR deficiency in SEC PDXs despite a high GIS, it was anticipated that PARPi monotherapy may have limited activity in these PDXs. Accordingly, combination therapies with known PARPi synergy in other cancers was tested in EC cell lines to build support for subsequent experiments. Previous studies have demonstrated that PARP inhibitors synergize with TOP1is in vitro and in vivo [25–29]. SN-38, the active metabolite of irinotecan, was tested as a single agent and combination with rucaparib in a panel of SEC cell lines. ARK-2 and SPAC1-S cell lines consistently showed that the impact of SN-38 on colony formation was increased when rucaparib was added. Formal mathematical analysis demonstrated a combination index (CI) < 1, indicative of synergy in these assays (Fig. 3A-D). Two other serous EC cell lines, ARK-1 and SPAC1-L, also showed enhanced effects on colony formation when rucaparib was added to SN-38, although greater variability in CI values was observed (Fig. 3E-F). To confirm that the observed effects could be attributed to increased apoptosis, flow cytometry was performed to determine the percentage of cells with extractable DNA or Annexin V staining, two indicators of apoptosis. Combination treatment was associated with increased apoptosis in all four cell lines, as illustrated in Fig. 4 for representative ARK-1 and SPAC1-L cells. Although ARK-2 cells demonstrated a measurable increase in apoptosis, the impact of combination treatment was less apparent (Supplemental Fig. 5A, B). Accordingly, the increased apoptosis in ARK-2 cells was confirmed using the Incucyte apoptosis assay, illustrated by a significant increase in caspase-3/7 activity after exposure to combination treatment (Supplemental Fig. 5C and Supplemental Methods).

### Rucaparib synergizes with SN-38 in SEC PDX tumors grown in 3D culture ex vivo

**Fig. 5:**
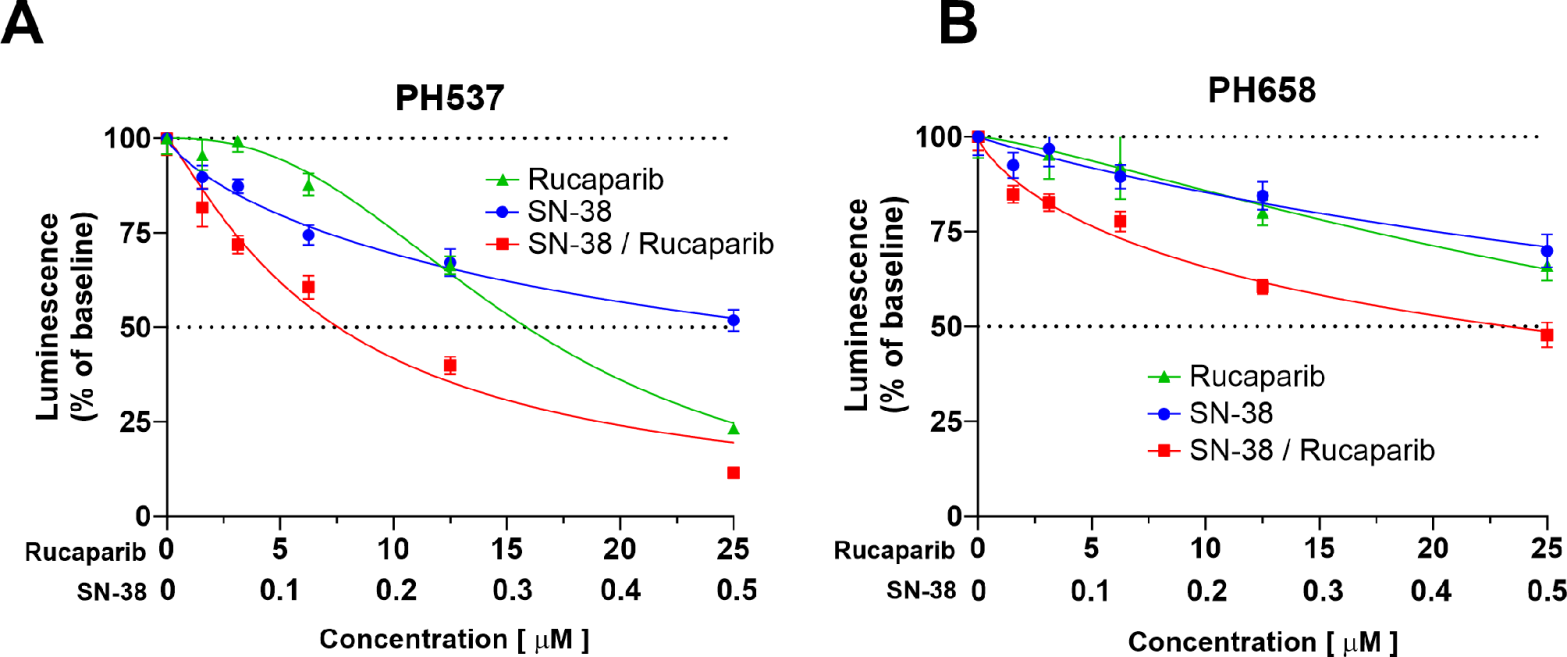
Activity of rucaparib and SN-38 in SEC tumors ex vivo. PDX tumors were exposed to rucaparib, SN-38, or the combination at the indicated concentrations. PH537 (**A**) and PH658 (**B**) are highlighted due to their markedly different GIS results

To further evaluate the SN-38 + rucaparib combination, the eight serous and two serous-like ECs were tested ex vivo. Each tumor was dissociated into small aggregates of cells for 3D culture on low-binding plates to promote three-dimensional growth. Across five rucaparib concentrations (1.56 to 25 µM), a dose-dependent decline in tumor cell viability was observed (Fig. 5 and Supplemental Fig. 6). Although the sample size (*n* = 10) is not sufficient to make conclusive statements about rucaparib single agent activity in 3D, the cell viability of two genomically disparate tumors, PH537 (GIS high) and U1561.019 (GIS low), dipped below 50% with rucaparib at the highest concentration (Fig. 5 and Supplemental Fig. 6). With the addition of SN-38 (0.1 to 0.5 µM), synergy was observed in PH537 (GIS 81) as well as PH658 (GIS 32) (Fig. 5). Similar synergy was observed in other PDX tumors except PH456 (GIS 54) and U1561.019 (GIS 28) at 0.5 FA (Supplemental Table 2), suggesting synergy could be achieved regardless of GIS.

**Fig. 6:**
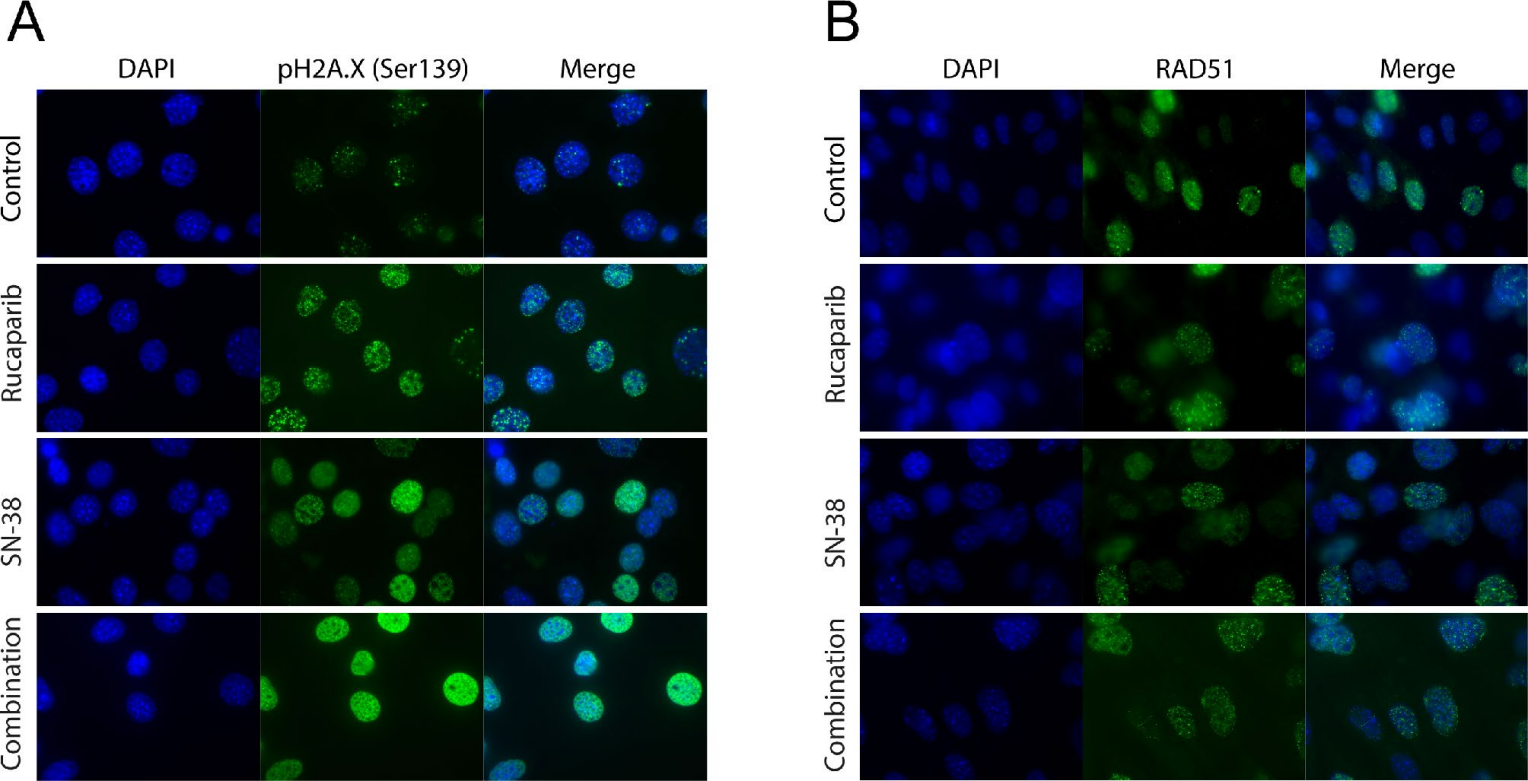
Immunofluorescence staining of DNA damage markers in PH537. Cells were treated with either vehicle, rucaparib, SN-38, or the combination. Foci were labeled with green fluorescence: (**A**) gamma H2AX (phospho-Ser139-H2A.X) or (**B**) RAD51. Nuclei were stained blue (DAPI)

To confirm that rucaparib, SN-38, and the combination could all induce double-strand DNA breaks in the GIS high PDX tumor PH537, phospho-H2A.X and RAD51 foci were examined. When PH537 cells were incubated on conventional 2D cell culture plates, treatment with rucaparib or SN-38 resulted in an increase in phospho-H2A.X foci, indicating induction of DNA double-strand breaks with either monotherapy (Fig. 6A). The combination induced even greater DNA damage, as indicated by the nearly confluent nuclear luminesces from coalescing foci. Moreover, the lack of RAD51 foci under the same conditions (Fig. 6B) was consistent with a loss of HR function, as shown in the irradiation studies (Fig. 2) for this PDX model.

### In vivo efficacy of rucaparib in combination with PLX038A

In further studies, five PDXs were treated with rucaparib monotherapy and the PLX038A/rucaparib combination in vivo. PDXs were selected to represent a wide range of GIS, 28 to 81. Although topotecan is more commonly used in gynecologic cancers than the SN-38 prodrug irinotecan, a novel pegylated formulation of SN-38 (PLX038A) has the ability to accumulate in tumors [32] and has a pharmacokinetic profile that could potentially minimize overlapping myelotoxicity with rucaparib: Low maximum plasma concentration and prolonged sustained plasma SN-38 above therapeutic thresholds [20, 32, 50]. To test the efficacy of rucaparib +/- PLX038A, PDX models were established intraperitoneally in female SCID-beige mice and when tumors reached a minimum size threshold, animals were randomized to receive either rucaparib 150 mg/kg daily, PLX038A 15 µmol/kg every two weeks, or the combination for a total of 8 weeks followed by one week of observation. Because tumor diameter accounts for only one dimension of growth, tumor size change over time was assessed weekly by transabdominal ultrasonography to determine response as described [34, 37, 43]. Tumor tissue (hypoechoic in mouse abdomen) can be discriminated from surrounding bowel/stool. Representative ultrasound images of PH537 show a single tumor mass with decreased tumor area and echogenicity after combination treatment, whereas saline-treated control and monotherapy-treated tumors grew without regression (Supplemental Fig. 7).

**Fig. 7:**
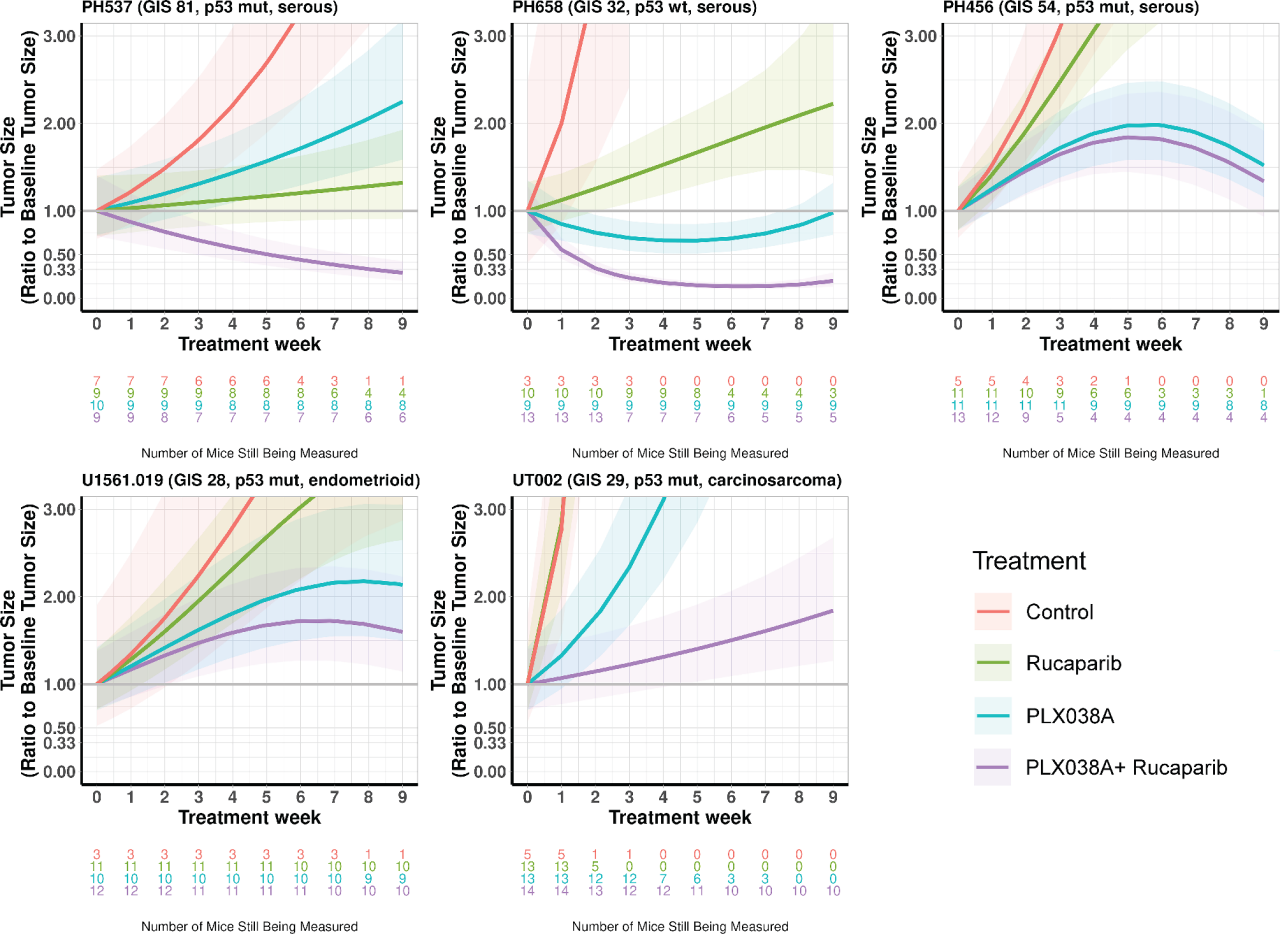
In vivo efficacy of PLX038A + rucaparib in SEC PDXs. Mice with measurable tumors were randomized to one of four groups: control (red), PLX038A every two weeks at 15 µmol/kg IP injection (blue), rucaparib 150 mg/kg daily gavage (green), or combination (purple) for 8 weeks, followed by one week of observation. Tumor size was assessed by weekly transabdominal ultrasound and normalized to the day zero starting size. P53 status by IHC is indicated as a wildtype (wt) or aberrant/mutated (mut) pattern. Tumor size trajectories are the average estimates computed from the statistical linear mixed effects models, relative to the arm-specific baseline estimate. Shading indicates 95% confidence intervals. The P values are provided in Supplementary Table 3. The number of mice under observation at each time point for each arm is indicated below the x-axis as a function of time, where text color indicates drug arm

Tumor sensitivity to monotherapy was different for rucaparib vs. PLX038A. Although none of the EC PDX models regressed with rucaparib monotherapy, two exhibited significant growth inhibition relative to controls: PH537 (GIS 81, *p* < 0.0001) and PH658 (GIS 32, *p* = 0.0365) (Fig. 7 and Supplemental Table 3). However, the doubling of tumor size in PH658 over 9 weeks would be consistent with relative rucaparib resistance. Whole genome sequencing performed on each of the 5 PDX models tested in vivo indicated that PH537 had a pathogenic mutation in a DNA repair gene (*PRKDC*, c.6151_6176del) while no DNA repair mutations were identified in PH658 or the other three PDXs. In contrast, PLX038A monotherapy demonstrated activity, albeit limited at a low dose, against all five SECs with statistically significant slowing of growth relative to control animals (Fig. 7 and Supplemental Table 3) and tumor shrinkage in one model.

Consistent with the ex vivo 3D culture results, the rucaparib/PLX038A combination was superior to PLX038A monotherapy in PH537, PH658, and UT002 (*p* < 0.001 vs. all treatment groups for each PDX). It is noteworthy that combination therapy resulted in marked regression of tumors below baseline sizes in two PDXs (PH537 and PH658). Moreover, tumor sizes frequently fell below detection limits by ultrasound at 9 weeks, with 4/8 mice bearing PH537 and 6/8 mice bearing PH658 lacking measurable disease, indicating marked sensitivity to the combination therapy.

### Rucaparib/SN-38 synergism in primary EC samples

**Fig. 8:**
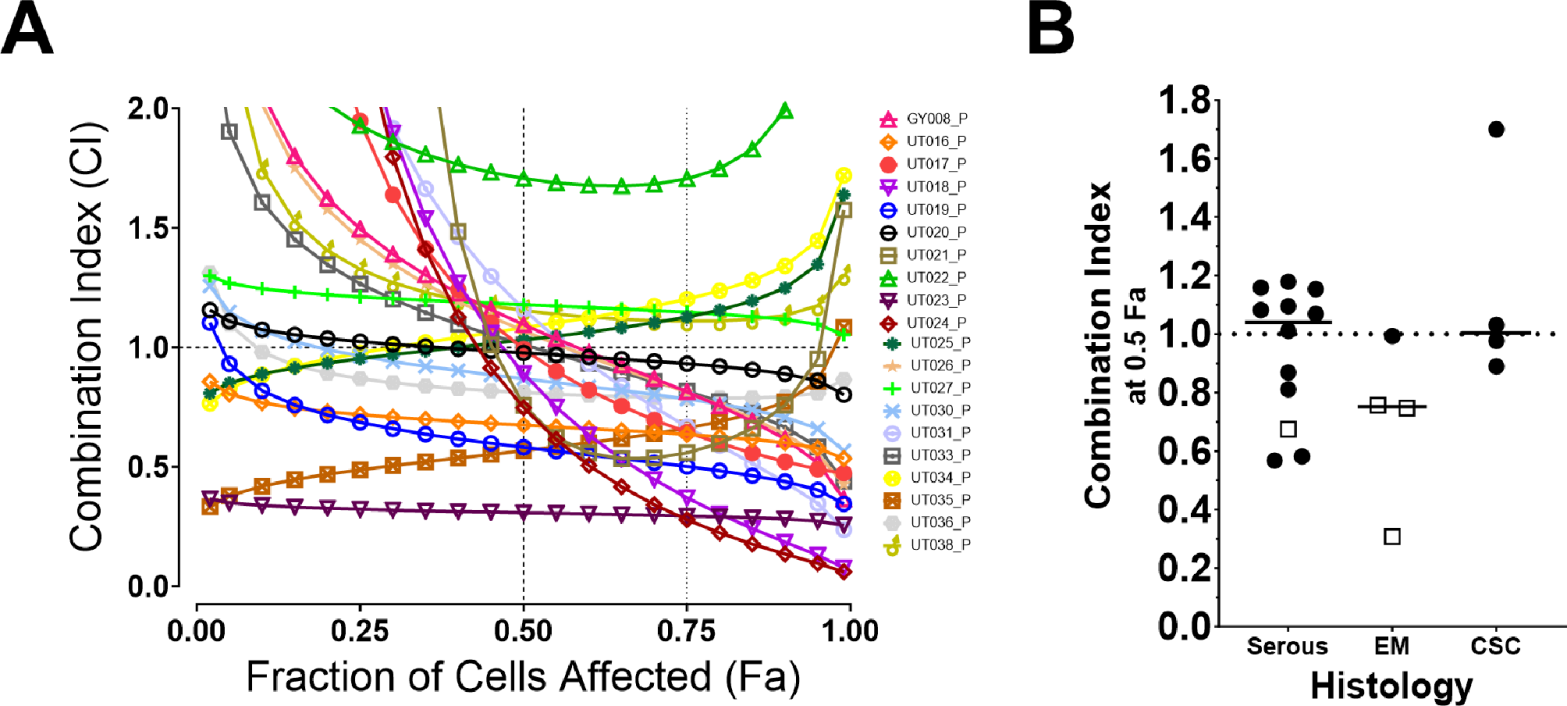
Primary patient tumors tested ex vivo for synergy. ECs were exposed to rucaparib, SN-38, or the combination at titrating concentrations. **A**) Six drug dilutions were used to generate the combination index (CI) curves using Calcusyn. **B**) The CI values at 0.5 fraction of cells affected (Fa) are plotted from panel A by histology: serous, endometrioid (EM), and carcinosarcoma (CSC). P53 aberrant ( ● ) and wildtype ( □ ) tumors are indicated

Although the in vitro and in vivo data support the efficacy of rucaparib + SN-38, primary patient tumors can provide an additional model system for preclinical evaluation of novel combinations. Ex vivo 3D cultures, also known as tumor organoids or microcancers, are more proximal to the patient than other model systems. To corroborate the cell line and PDX data, primary patient tumors were also assayed for effects of the individual agents and combination. ECs collected fresh from surgical resections or imaging-guided biopsies were processed immediately for ex vivo 3D culture. Because IHC for p53 was not universally performed at the time this study began, the only inclusion criteria were a biopsy-proven SEC (*n* = 12), high grade endometrioid EC (*n* = 4), or carcinosarcoma (*n* = 4) (Supplemental Table 4). Synergy (combination index < 1) was observed in 11 of 20 (55%) samples at 0.5 FA (Fig. 8A, B), and individual primary tumor cell growth inhibition curves were also plotted (Supplemental Fig. 8). Interestingly, all samples with endometrioid histology and wildtype-pattern p53 exhibited synergy (Fig. 8B and Supplemental Table 3), suggesting the synergy may not be limited to serous-like cancers.

## Discussion

SEC is associated with chemotherapy resistance and a high risk for recurrence regardless of clinical stage, making improved SEC therapeutics a large unmet need. To facilitate the search for improved therapies, we established 30 EC PDX models, including 10 from serous or serous-like ECs, and then assessed their response to an emerging therapy (PLX038A as monotherapy and in combination with rucaparib). Previous studies revealed that a subset of serous or serous-like ECs exhibit evidence of HR DNA repair deficiency, raising the possibility that PARP inhibitors might have activity in this disease. To address this possibility, we assessed a trimodal genomic instability score (GIS) based on telomeric allelic imbalance, large state transitions, and loss of heterozygosity in EC PDXs. However, GIS did not correlate with HR function and rucaparib had limited activity in vivo. Importantly, the combination of rucaparib/SN-38 was more effective than monotherapy and response was not dependent on HR deficiency.

Genomic methods to predict HR deficiency in ovarian cancer have relied on a trimodal score [15, 17] or LOH alone [50] but it remains unclear if such methods are applicable to EC. In preclinical studies, the largest report of a trimodal GIS in SECs indicated that 10 of 19 sequential tumors (53%) had scores ≥42 [53], comparable to our observation of 62.5% (5 of 8). However, no functional assays to confirm HR deficiency by RAD51 foci formation assay or in vivo PARPi response were performed. Another study using a trimodal GIS in four serous ECs included only GIS < 42, making it difficult to assess if a high GIS could enrich for PARPi sensitivity [54]. While we observed a GIS score > 42 in 5 of 8 SEC PDX models, evidence for functional loss of HR was limited to 1 of 5 PDX models, possibly reflecting the fact that genomic changes leading to a high GIS score reflect the historical inability of a tumor to repair DNA but persist if cancers regain their HR repair capacity. Indeed, it is well known that ovarian cancers with germline or somatic *BRCA1/2* mutations and GIS can undergo reversion mutations to restore BRCA1/2 expression and restore HR proficiency without altering the high GIS [55, 56]. Other ovarian cancers undergo epigenetic reactivation of HR proteins [56, 57]. On the other hand, there may be occasions where a GIS might not be sensitive enough to detect all HR deficient tumors. To overcome such limitations and minimize the risk for false negative results, perhaps alternative methods should be used to predict HR deficiency. Examples include mutational [58] or transcriptional [59, 60] signatures. Perhaps a multiomics approach would also be helpful. Regardless, none of the 5 tested SEC PDX models exhibited regression with PARPi monotherapy. Thus, the experiments presented suggest that a high GIS in SEC PDXs does not correlate with loss of HR function and a high GIS does not portend sensitivity to a PARPi.

Previous clinical studies testing the efficacy of a PARPi in EC have not focused solely on SECs, but have evaluated the correlation between GIS and clinical response. For instance, olaparib monotherapy in recurrent EC patients (NRG-GY012) resulted in a 12.5% (4/32) objective response rate and median progression free survival of only 2 months [61]. Although the patient population was not limited to serous/serous-like ECs, the majority of tumors were P53 aberrant. Importantly, no associations were found between olaparib response and mutations in HR DNA repair genes or LOH as a measure of HR deficiency. Similarly, a separate clinical study of niraparib in predominately serous EC recently reported only a 4% (1/25) objective response rate and found no correlation with aberrations in HR DNA repair genes [62]. These clinical trial outcomes are consistent with our rucaparib monotherapy results in five serous or serous-like EC PDX models (GIS diverse), which revealed only stable disease (PH537) or slowed progression (PH658) as the best response, without regression on rucaparib monotherapy.

PLX038A is a pegylated SN-38 prodrug that is designed to substantially prolong the half-life of SN-38 without reaching high serum peak levels [20, 32, 50]. This novel formulation should help to minimize toxicity and optimize efficacy when combined with PARPis. This agent demonstrated monotherapy activity in all five SEC PDXs. Moreover, when PLX038A was combined with rucaparib, mice tolerated the treatment, and the combination was more effective than either agent alone in 3 of 5 PDX models. Importantly, the improved efficacy of this combination in vitro (cell lines), ex vivo (primary patient and PDX), and in vivo appears to be independent of genomic instability or HR deficiency, which suggests it might have broad activity in ECs. Without clear associations between genetic mutations and response to rucaparib or combination therapy, an alternative predictive biomarker of response warrants further investigation. Interestingly, the observation that rucaparib and SN-38 are synergistic in non-serous and p53 wildtype primary patient tumors (Fig. 8B), raises the possibility of activity with this combination in the “no specific molecular profile” (NSMP) of EC, which is the largest molecular subtype. Regardless, these data support further preclinical and possible clinical development of PLX038A + rucaparib in EC. Accordingly, we are awaiting the recommended phase 2 dose from an ongoing phase 1 trial with this combination (NCT04209595).

## Conclusions

In summary, serous or serous-like EC PDX models and EC patient explants are sensitized to the combination of SN-38 and rucaparib. However, the in vivo response to single agent rucaparib suggests that a PARPi might only delay EC progression, regardless of GIS. Although an improvement in progression-free survival is still clinically meaningful, the studies presented herein would suggest that PARP inhibitor monotherapy might not be particularly efficacious in EC. Instead, as novel formulations TOP1 inhibitors such as PLX038A are developed clinically, EC should be included in the development plan.

## Electronic supplementary material

Below is the link to the electronic supplementary material.

Supplementary Material 1: Supplemental Fig. 1. Patient derived xenograft engraftment rate over time, where “success” indicates successful engraftment in at least one mouse and “failed” indicates failed engraftment. Time to engraftment and engraftment rate were determined using a cumulative incidence approach to account for models still under observation for determination of engraftment. Supplemental Fig. 2. Functional assessment of homologous recombination (HR) activity in PEO1 and PEO4 cells. All representative images were captured at 100x magnification. Supplemental Fig. 3. Immunohistochemistry showing p53 staining pattern. Tumors studied in vivo are shown. All representative images were captured at 40x magnification. Scale bar shows 50 μm. Supplemental Fig. 4. Histologic similarities between patients and corresponding patient derived xenograft (PDX) tumors. Representative hematoxylin and eosin (H&E), p53 expression in PDX EC models showed conserved morphology, (20X). Scale bar, 100 μm. Supplemental Fig. 5. Induction of apoptosis in ARK-2 cell lines with SN-38 and Rucaparib. A) cells were cultured with continuous exposure to the indicated drug concentrations for 4 days, stained with propidium iodide in sodium citrate (A), and subjected to flow microfluorimetry. Error bars indicate mean SEM of 3–4 independent experiments. B), combination index (CI) for the SN-38 + rucaparib drug combination. Different shapes indicate results from each of 3–4 independent experiments. C), Caspase release assay showing the amount of caspase-3/7 induction per well with SN-38(10nM), rucaparib(100nM) or combination treatment in ARK-2 cells using Incucyte Live-Cell analysis. ANOVA with Tukey’s multiple comparisons test *p* = 0.0003 (*) or 0.0002 (**). Supplemental Fig. 6. Activity of Rucaparib and SN-38 in SEC PDXs ex vivo. PDX tumors were exposed to rucaparib, SN-38, or the combination at the indicated concentrations. Cell viability was measured in luminescence and normalized to untreated controls. Supplemental Fig. 7. Representative serial transabdominal ultrasound images from PH537 PDX showing assessment of tumor (dotted outline) change over eight weeks of treatment. White scale bar is 5 mm. The circumferential dotted line outlines each tumor and shows the measured cross-sectional area. Supplemental Fig. 8. Activity of Rucaparib and SN-38 in SEC tumors ex vivo. Fresh primary patient tumor cells were exposed to rucaparib, SN-38, or the combination at the indicated concentrations. Cell viability was measured in luminescence and normalized to untreated controls.

## Data Availability

The datasets used and/or analyzed during the current study are available from the corresponding author on reasonable request.

## Abbreviations

SEC: Serous Endometrial Cancer
EC: Endometrial Cancer
PDX: Patient-Derived Xenograft
HR: Homologous Recombination
PARP: Poly(ADP-Ribose) Polymerase
PARPi: Poly(ADP-Ribose) Polymerase Inhibitor
ORR: Objective Response Rate
LOH: Loss Of Heterozygosity
TOP1: Topoisomerase 1
TOP1is: Topoisomerase 1 Inhibitors
TDP1: Tyrosyl-DNA Phosphodiesterase 1
PLX038A: PEGylated SN-38
IRB: Institutional Review Board
WGS: Whole Genome Sequencing
GGPS: GenomeGPS
GIS: Genomic Instability Score
CNV: Copy Number Variation
AAC: Alternative Allele Concentration
LST: Large Scale Transitions
TAI: Telomeric Allele Imbalance
FBS: Fetal Bovine Serum
IHC: Immunohistochemistry
LCWGS: Low-Coverage Whole Genome Sequencing
H&E: Hematoxylin and Eosin
NSMP: No Specific Molecular Profile
