# Supplementary material for: Antitumor activity of rucaparib plus PLX038A in serous endometrial carcinoma
Source: J Exp Clin Cancer Res. 2025 May 19;44:150. doi: 10.1186/s13046-025-03406-7 (PMC12087071; doi:10.1186/s13046-025-03406-7)
Supplement: Supplementary file 1 — Supplementary Material 1: Supplemental Fig. 1. Patient derived xenograft engraftment rate over time, where “success” indicates successful engraftment in at least one mouse and “failed” indicates failed engraftment. Time to engraftment and engraftment rate were determined using a cumulative incidence approach to account for models still under observation for determination of engraftment. Supplemental Fig. 2. Functional assessment of homologous recombination (HR) activity in PEO1 and PEO4 cells. All representative images were captured at 100x magnification. Supplemental Fig. 3. Immunohistochemistry showing p53 staining pattern. Tumors studied in vivo are shown. All representative images were captured at 40x magnification. Scale bar shows 50 μm. Supplemental Fig. 4. Histologic similarities between patients and corresponding patient derived xenograft (PDX) tumors. Representative hematoxylin and eosin (H&E), p53 expression in PDX EC models showed conserved morphology, (20X). Scale bar, 100 μm. Supplemental Fig. 5. Induction of apoptosis in ARK-2 cell lines with SN-38 and Rucaparib. A) cells were cultured with continuous exposure to the indicated drug concentrations for 4 days, stained with propidium iodide in sodium citrate (A), and subjected to flow microfluorimetry. Error bars indicate mean SEM of 3–4 independent experiments. B), combination index (CI) for the SN-38 + rucaparib drug combination. Different shapes indicate results from each of 3–4 independent experiments. C), Caspase release assay showing the amount of caspase-3/7 induction per well with SN-38(10nM), rucaparib(100nM) or combination treatment in ARK-2 cells using Incucyte Live-Cell analysis. ANOVA with Tukey’s multiple comparisons test p = 0.0003 (*) or 0.0002 (**). Supplemental Fig. 6. Activity of Rucaparib and SN-38 in SEC PDXs ex vivo. PDX tumors were exposed to rucaparib, SN-38, or the combination at the indicated concentrations. Cell viability was measured in luminescence and normalized to untreated [file 13046_2025_3406_MOESM1_ESM.docx]

**Supplementary Material to**

**Antitumor activity of rucaparib plus PLX038A in serous endometrial carcinoma**

Xiaonan Hou, Valentina Zanfagnin, Conway Xu, Erik Jessen, Yuanhang Liu, Chen Wang, Yajue Huang, Shaun D. Fontaine, Daniel V. Santi, Gerardo Colon-Otero, Sara E. Gill, Gretchen E. Glaser, Kristina A Butler, Jamie N. Bakkum-Gamez, Sean Dowdy, Ann L. Oberg, Melissa C. Larson, Hunter J. Atkinson, Laura N. Duffield, Kevin L. Peterson, Scott H. Kaufmann, and S. John Weroha

**Supplementary Methods**

***Derivation of genomic instability score and variant calling***

The analysis of whole genome sequencing (WGS) data of PDXs was conducted using the Mayo Bioinformatics in-house pipeline named GenomeGPS (GGPS), a comprehensive toolset for the alignment and analysis of DNA sequencing data. GGPS processes custom capture panels, exome, and whole genome technologies through various stages, including alignment, variant calling, quality control, and copy number variation (CNV) modules. The pipeline utilizes DNA FASTA files, a flag indicating whether reads are paired or single end, groups of samples if somatic calling is needed, and contextual inputs such as working directories. Murine reads were removed prior to GGPS processing by using bwa v0.7.10 to align all reads to either the human (hg38) or mouse (mm10) genomes. These alignments were then processed with the R package Xenofilter, which flags reads as ambiguous, human-specific, mouse-specific, or aligning to both genomes. Only human-specific and ambiguous reads were retained for subsequent steps. After murine filtering, this WGS dataset had reasonable intermediate coverage to the human genome (11.1x averaged base-pair coverage and 9x median base-pair coverage) for research variant calling and CNV analytics. BAM files containing human genome reads were realigned using GATK IndelRealigner, followed by splitting the BAM files by chromosome. The realigned BAM files were then recalibrated and merged into a final BAM file for each sample. ***For variant calling***, HaplotypeCaller was used with a minimum allele frequency of 0.2. Somatic variant calling was conducted on the realigned BAM files using GATK Mutect2 in tumor-only mode, with GNOMAD employed as the reference database. The ***genomic instability score (GIS)*** was computed in four steps:

1. **CNV Quantification and Segmentation:** Copy number variants (CNVs) for whole genome samples were quantified using the tool Wandy, which assesses deviations from the median coverage for 10kb genome bins. To minimize technical variation due to GC content differences, GC correction was applied. Regions consistently deviating from the median in normal CNV samples, whether positive, negative, or alternating, were flagged as difficult to quantify and excluded from the analysis. The bin-level normalized coverage was converted to a log fold change based on the median normalized coverage for each sample. Similar adjacent bins were combined using a regression decision tree algorithm optimized for analyzing whole genome samples with 10kb bin coverages, scaled by chromosome size, to form the final CNV segmentation calls.
2. **Alternative Allele Concentration Quantification and Segmentation:** Regions of allelic imbalance were identified by backfilling common dbSNP variants and detecting deviations in the absolute change of alternative allele concentration (AAC) of all heterozygous SNPs in genomic bins from the expected values. GATK UnifiedGenotyper determined the allele counts for 6.23 million dbSNP variants across all autosomal chromosomes. A bin size of 1Mb with a 200Mb rolling window was used due to the distribution and quantity of dbSNP variants. For low and intermediate coverage samples (1x-10x), the bin size was increased to 2.5Mb. SNPs with allele concentrations between 0.05 and 0.95 were classified as non-homozygous, and for each bin, the average absolute deviation of AAC values from the median value for non-homozygous SNPs (~0.46-0.49 due to reference allele bias) was calculated. This allele imbalance metric was converted to a log fold change using the median value among all bins. Similar adjacent bins were joined using the regression decision tree algorithm described in the CNV calling section.
3. **Allele-specific CNV Calling:** An algorithm combined the CNV segmentation and AAC segmentation to create allele-specific CNV calls. The CNV calls formed the basis of these calls, with the log fold change being converted to estimated copy number. Since the samples were from PDX models that had undergone murine subtraction, the tumors were assumed to be relatively pure. Tumor purity was estimated using two methods: 1) histogram of all segmentation calls to estimate purity from the peak deviation of the +1/-1 copy number change peaks from the expected 0.58/-1 values; and 2) allele imbalance of deletion and duplication regions and the deviation in magnitudes from the expected 0/1 and 0.33/0.66 shifts in heterozygous allele frequencies. The estimated copy numbers were adjusted for estimated tumor purity. The AAC segment calls and magnitudes were used to assign copy numbers to pseudo-alleles (default A). For deletions, a region with imbalanced alleles resulted in copy numbers assigned to allele A. Balanced deletions, indicative of tetraploid tumors, required modifying total copy number counts by a factor of two. For duplications and amplifications, imbalanced AAC segmentation resulted in all but one copy number assigned to allele A (CN-1 allele A, 1 allele B). Balanced AAC segmentation assigned equal estimated copy numbers to alleles A and B (CN/2 allele A, CN/2 allele B), with any odd copy number assigned to allele A. For CNV-normal segments, imbalanced AAC segmentation assigned all copy numbers to allele A as a copy-neutral loss of heterozygosity. CNV segments with differing imbalanced or balanced AAC assignments were appropriately split.
4. **HRD score aggregations:** Allele-specific CNV calls, denoted as estimated copy numbers from allele A and allele B, were provided to the R package ScarHRD (<https://www.nature.com/articles/s41523-018-0066-6>). ScarHRD, developed to calculate HRD scores from microarray data, was repurposed for calculating HRD from whole genome sequencing allele-specific CNV calls. ScarHRD counts the total number of events meeting the following criteria: 1) Loss of Heterozygosity (LOH), the number of LOH regions greater than 15Mb and smaller than an entire chromosome; 2) Large Scale Transitions (LST), the number of adjacent chromosomal breaks of at least 10Mb with less than 3Mb separation; and 3) Telomeric Allele Imbalance (TAI), the number of regions with allele imbalance encompassing the chromosome ends. The resulting sub-scores were summed to obtain a final HRD score for each sample.

**Caspase 3/7 activity assay by Incucyte Live-Cell analysis**

ARK2 cells were seeded in 96-well plates were treated with SN-38, rucaparib or combination in the presence of Incucyte Caspase-3/7 Green Apoptosis Assay Reagent (#4440, Sartorius) following the manufacturer’s protocol. Three images per well were acquired every 2hours for total 72hours with the adequate light channel following the manufacturer’s recommendations. Green-fluorescence staining counts were used to measure apoptosis. Data were plotted in GraphPad Prism as the mean +/- SEM of replicate wells.

***Statistical analysis of PDX efficacy studies***

Repeated measures implemented via linear mixed effects models were used to compare tumor growth trajectories between arms on the natural log scale separately for each PDX (PH456, PH537, PH658, U1561.019, and UT002) as previously described [1]. The treatment phase (weeks 0-8) was modeled with day (centered by subtracting the duration of the treatment phase divided by 2), an indicator of treatment arm, and the 2-way interaction as fixed effects. A quadratic centered day effect was included to accommodate curvature in the trajectories for PH456, PH658, and U1561.019, and a quadratic centered day by treatment arm interaction was needed for PH658. By centering the day variable, the intercept estimate can be interpreted as an estimate of the area under the curve. Spatial (power) correlation structure was used to account for the correlation between repeated observations with day (uncentered). Mean values in figures are estimates from these models. Error bars in figures represent 95% confidence intervals. A single 2-df hypothesis test of coincident curves (accounts for both mean and slope) was performed to assess differences between drug arms. If the coincident curve test was statistically significant, the intercepts and slopes were compared to determine the nature of the differences. Two mice in PH658 provided data for both the control and combination arms which is not accurately captured by the mixed effect model. While the statistical analysis in this case does not accurately reflect the true sample size, sensitivity analyses demonstrated conclusions were the same with and without this data. Thus, all data for these mice were included for the most precise estimates. Analyses were performed in SAS (SAS Institute Inc. Cary, NC, USA) and R software (R 4.1.0; R Foundation for Statistical Computing, Vienna, Austria).

**Supplementary References**

1. Oberg, A.L., et al., Statistical analysis of comparative tumor growth repeated measures experiments in the ovarian cancer patient derived xenograft (PDX) setting. Scientific Reports, 2021. **11**(1).

**
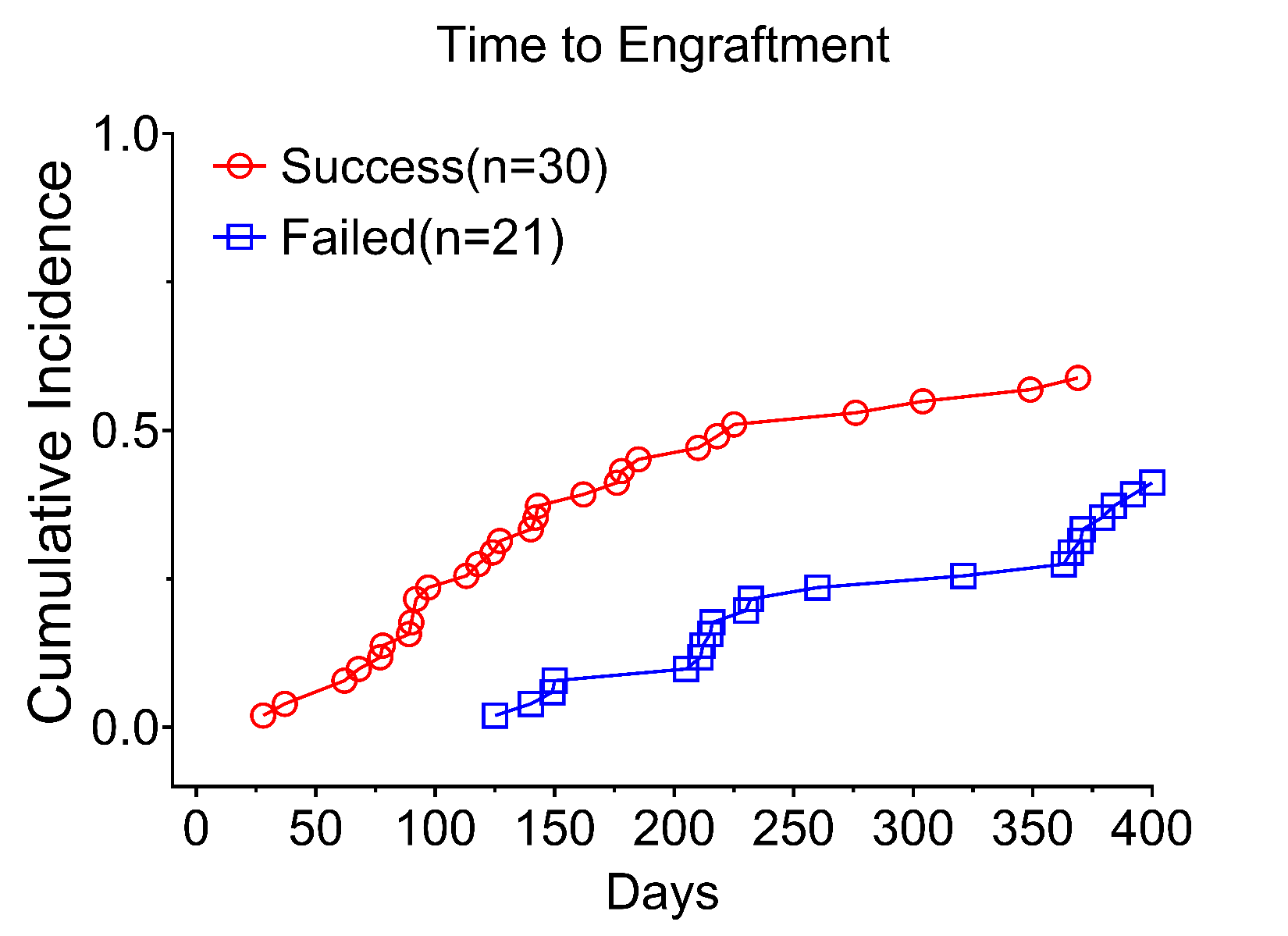
**

**Supplemental Figure 1.** Patient derived xenograft engraftment rate over time, where “success” indicates successful engraftment in at least one mouse and “failed” indicates failed engraftment. Time to engraftment and engraftment rate were determined using a cumulative incidence approach to account for models still under observation for determination of engraftment.

**
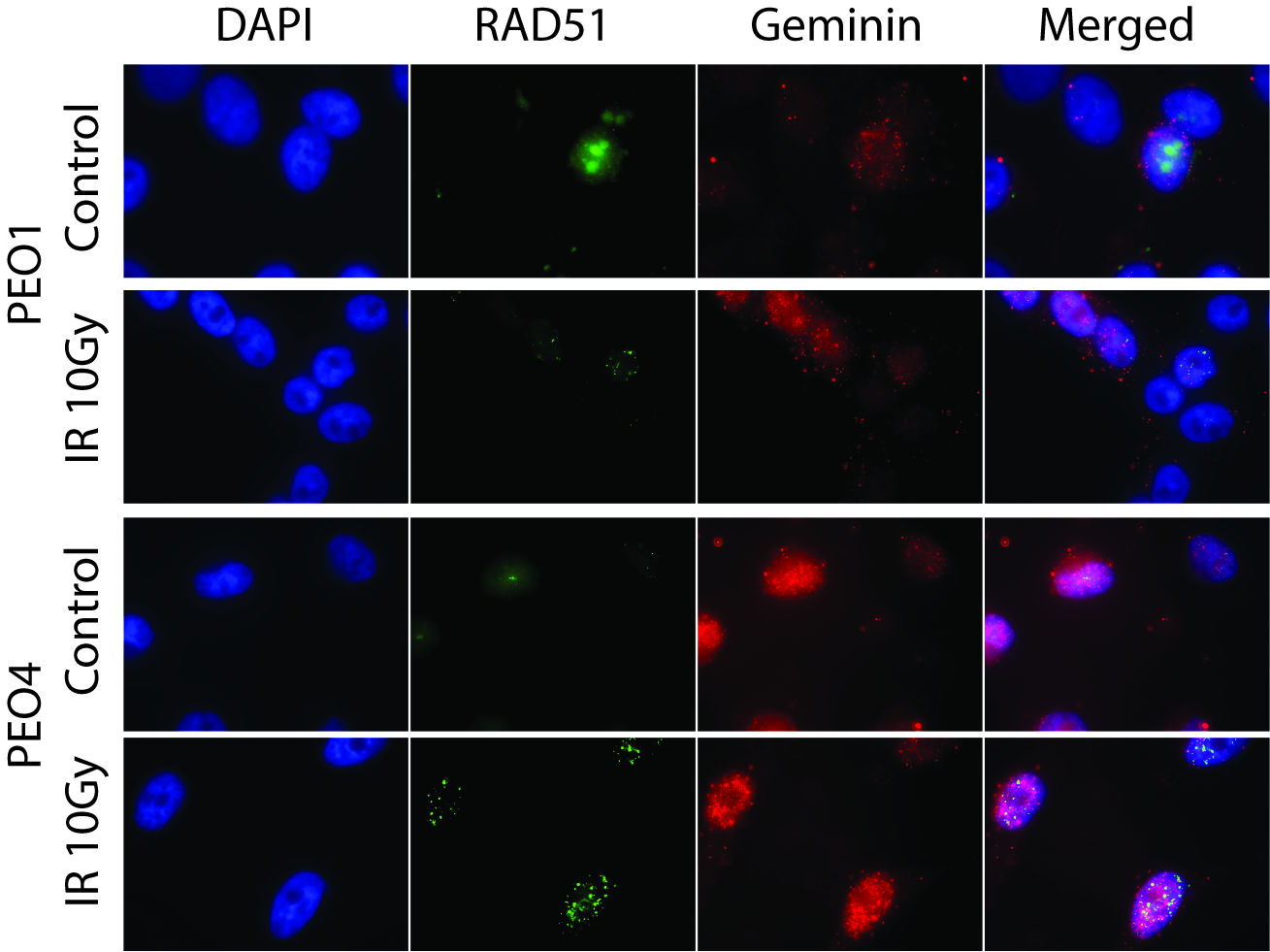
**

**Supplemental Figure 2.** Functional assessment of homologous recombination (HR) activity in PEO1 and PEO4 cells. All representative images were captured at 100x magnification.

**
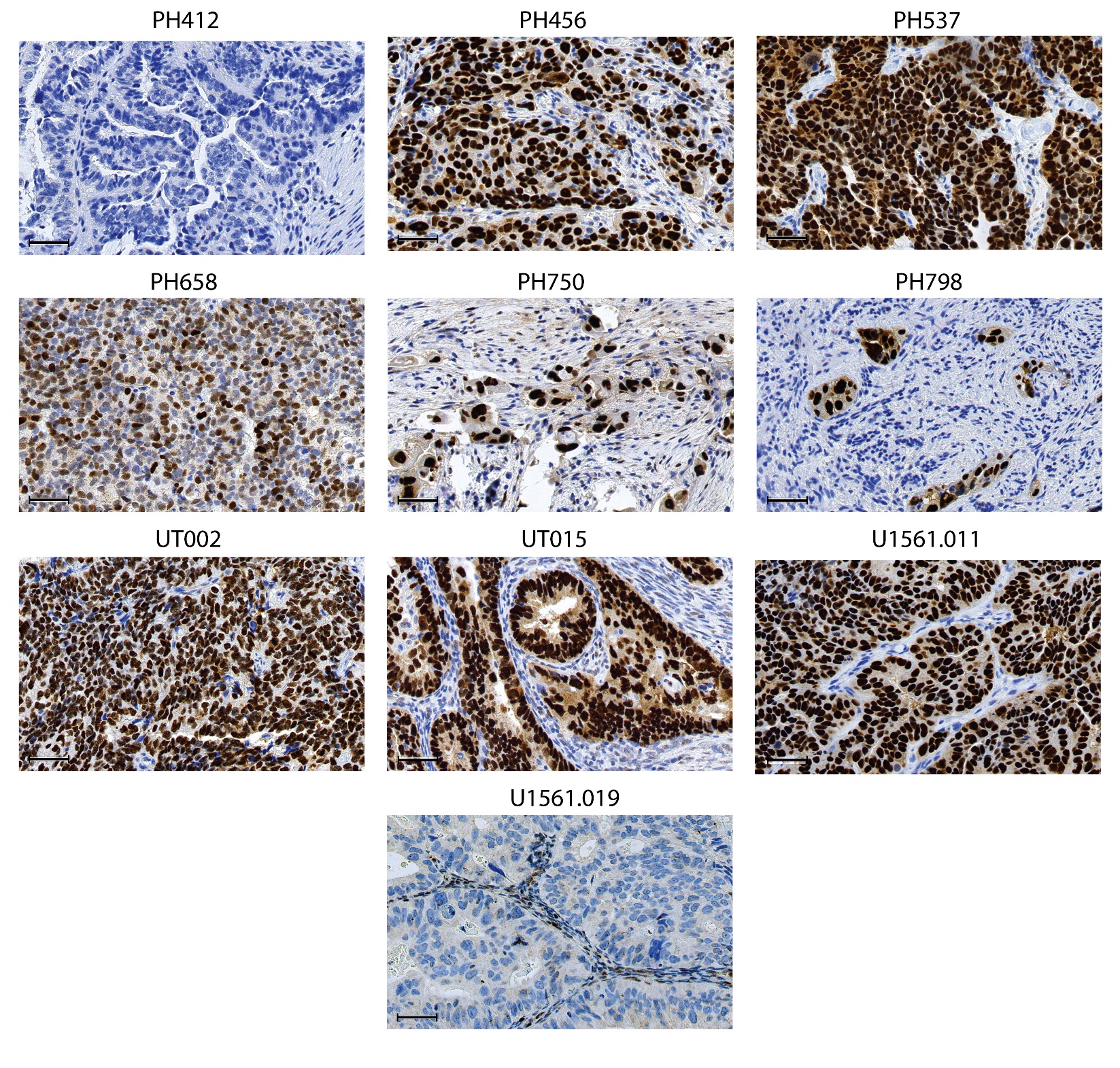
**

**Supplemental Figure 3. Immunohistochemistry showing p53 staining pattern.** Tumors studied *in vivo* are shown. All representative images were captured at 40x magnification. Scale bar shows 50 µm.

**
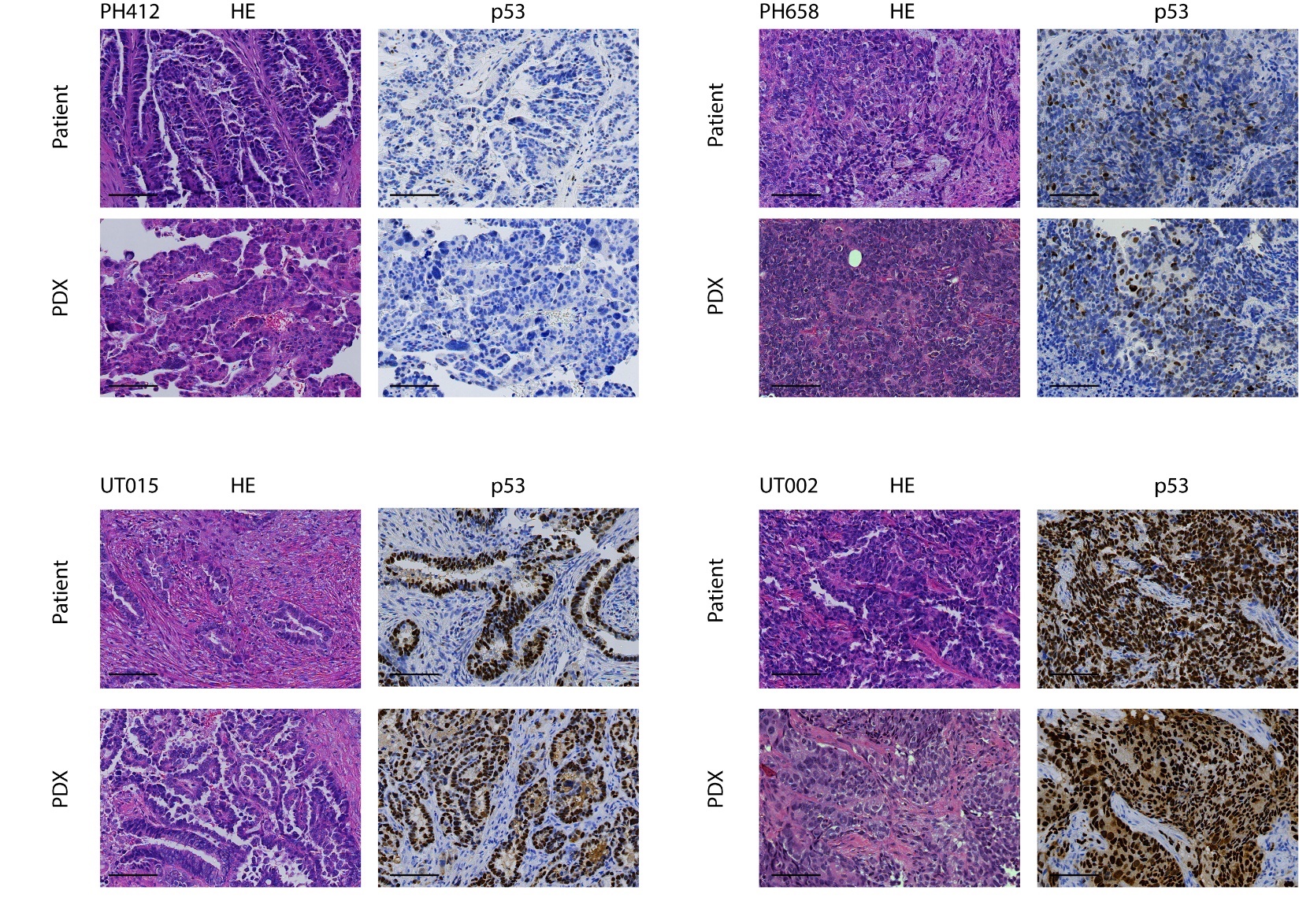
Supplemental Figure 4. Histologic similarities between patients and corresponding patient derived xenograft (PDX) tumors.** Representative hematoxylin and eosin (H&E), p53 expression in PDX EC models showed conserved morphology, (20X). Scale bar, 100 μm.


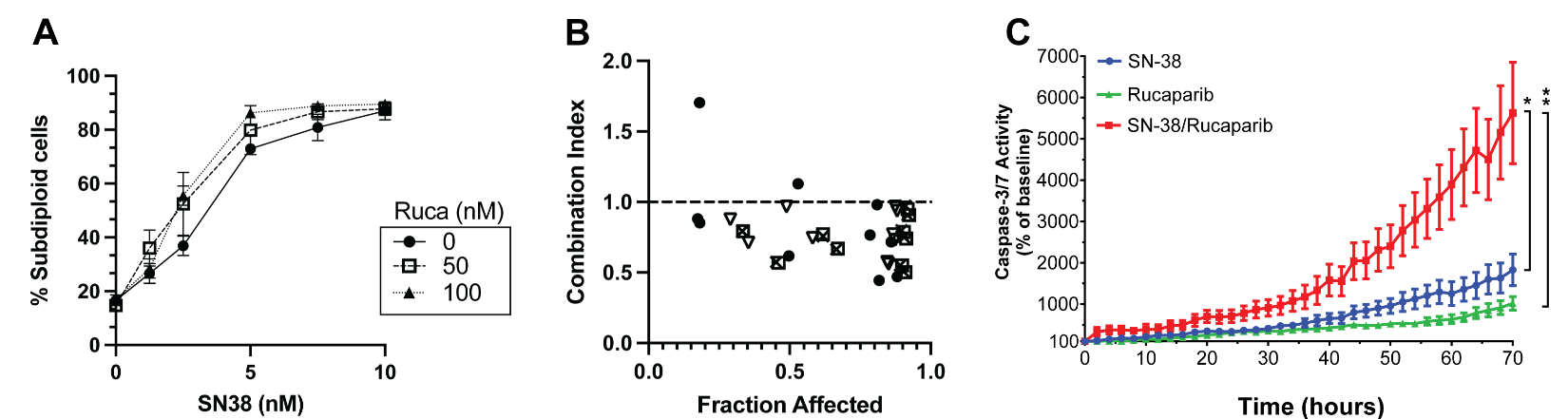


**Supplemental Figure 5. Induction of apoptosis in ARK-2 cell lines with SN-38 and rucaparib. A),** cells were cultured with continuous exposure to the indicated drug concentrations for 4 days, stained with propidium iodide in sodium citrate **(A),** and subjected to flow microfluorimetry. Error bars indicate mean $\pm$ SEM of 3-4 independent experiments. **B),** combination index (CI) for the SN-38 + rucaparib drug combination. Different shapes indicate results from each of 3-4 independent experiments. **C)**, Caspase release assay showing the amount of caspase‐3/7 induction per well with SN-38(10nM), rucaparib(100nM) or combination treatment in ARK-2 cells using Incucyte Live-Cell analysis. ANOVA with Tukey’s multiple comparisons test p = 0.0003 (*) or 0.0002 (**).

**Supplemental Figure 6. Activity of rucaparib and SN-38 in SEC PDXs *ex vivo***. PDX tumors were exposed to rucaparib, SN-38, or the combination at the indicated concentrations. Cell viability was measured in luminescence and normalized to untreated controls.


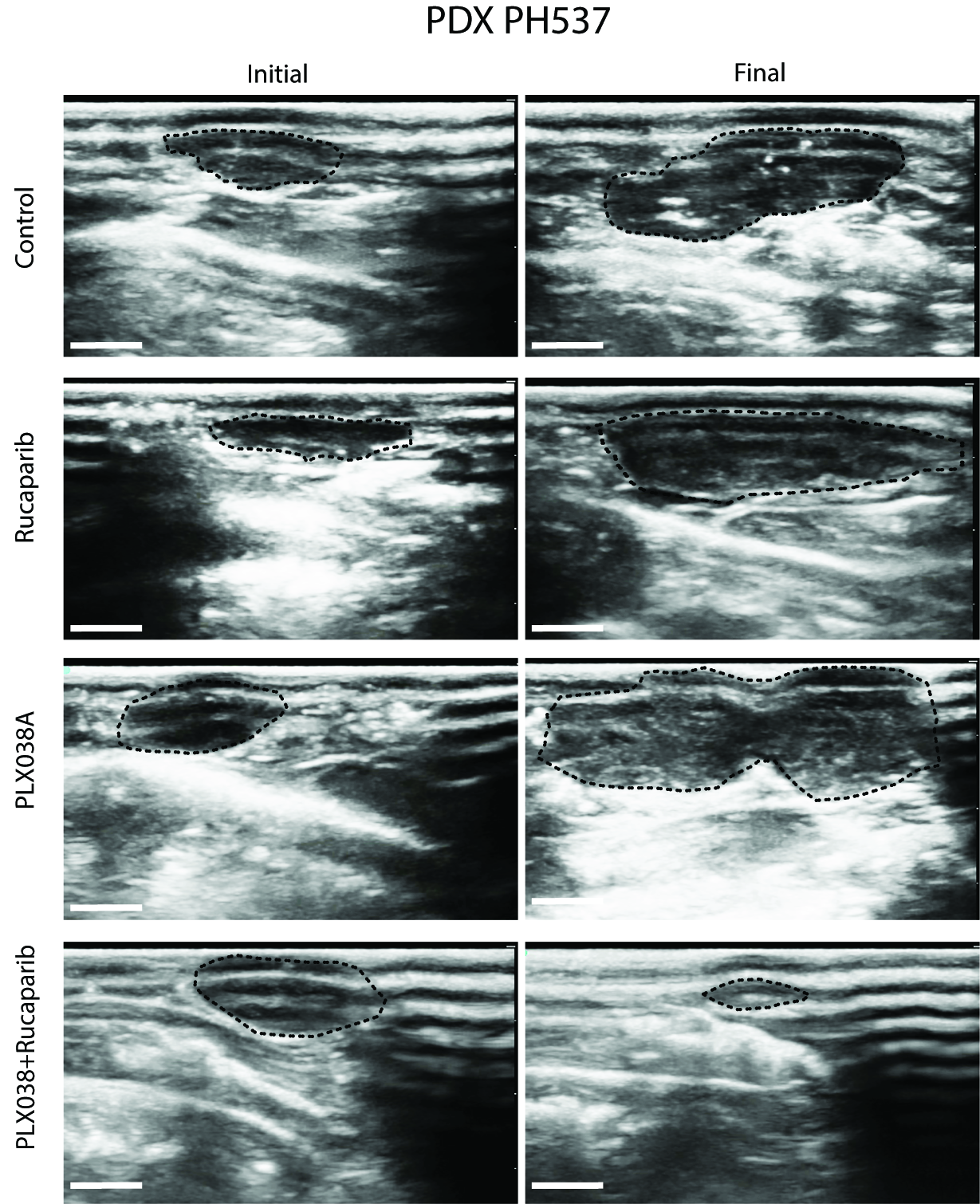


**Supplemental Figure 7.** Representative serial transabdominal ultrasound images from PH537 PDX showing assessment of tumor (dotted outline) change over eight weeks of treatment.  White scale bar is 5 mm. The circumferential dotted line outlines each tumor and shows the measured cross-sectional area.


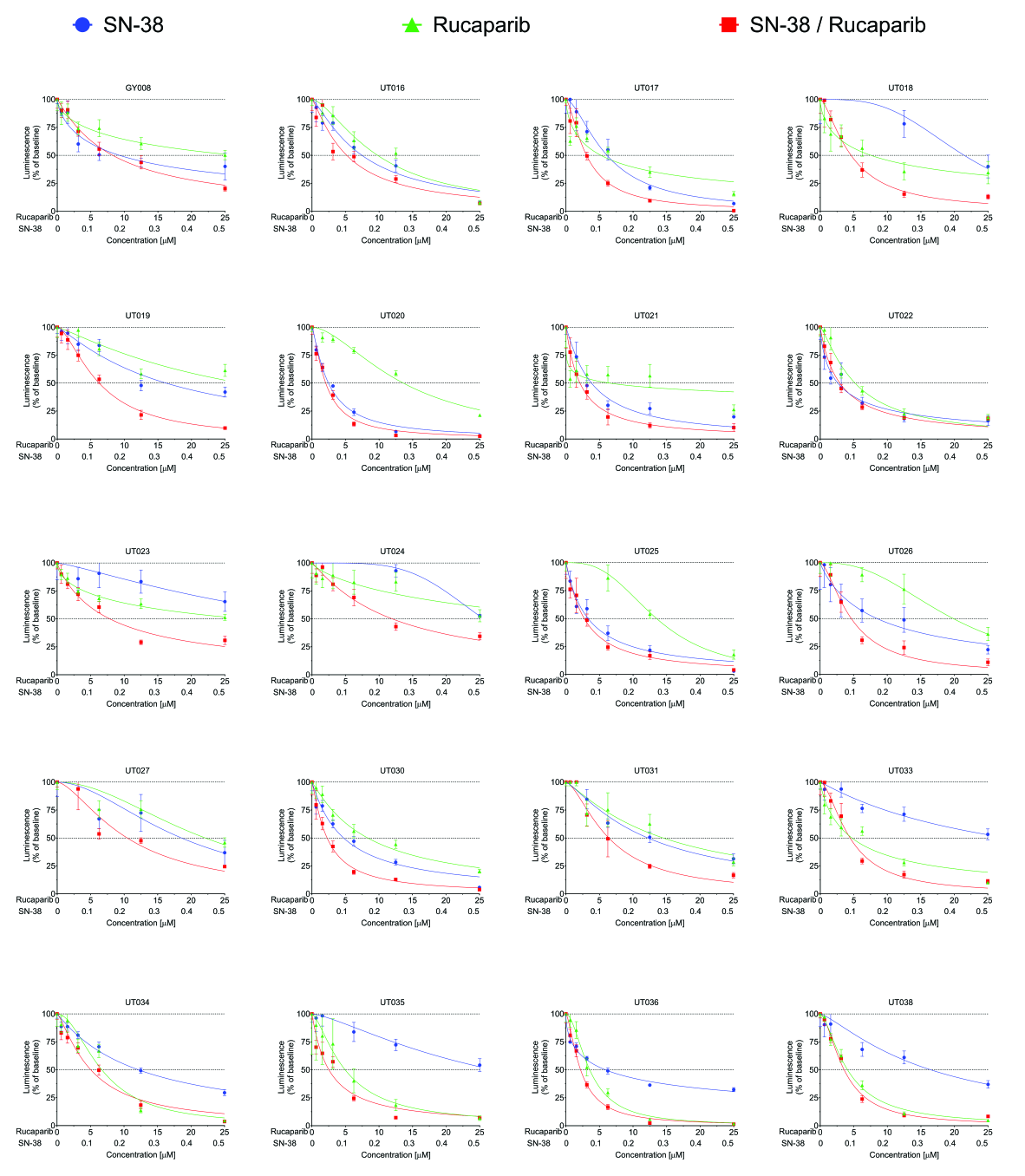


**Supplemental Figure 8. Activity of rucaparib and SN-38 in SEC tumors *ex vivo*.** Fresh primary patient tumor cells were exposed to rucaparib, SN-38, or the combination at the indicated concentrations. Cell viability was measured in luminescence and normalized to untreated controls.

**Supplemental Table 1. Clinical characteristics of the 10 tumors analyzed for genomic instability and homologous recombination deficiency**. Staging was based on FIGO 2009 since all tumors were collected prior to the 2023 update. High grade is considered 3 or 4. All carcinosarcomas are considered high grade and a numerical value for grade is not reported. Systemic or radiation therapy for endometrial cancer, if any, is only reported if it was administered prior to tumor collection.

|  | **PH412** | **PH456** | **PH537** | **PH658** | **PH750** | **PH798** | **UT002** | **UT015** | **U1561.011** | **U1561.019** |
| --- | --- | --- | --- | --- | --- | --- | --- | --- | --- | --- |
| **Histology** | Serous | Serous | Serous | Serous | Serous | Serous | Carcinosar-coma | Serous | Serous | Endometri-oid |
| **Grade** | High | High | High | High | High | High | n/a | High | High | High |
| **Stage** | IV | IV | IV | IIIC2 | IV | IVB | IB | IIIC | II | IVB |
| **p53 (IHC)** | Aberrant | Aberrant | Aberrant | Normal | Aberrant | Aberrant | Aberrant | Aberrant | Aberrant | Aberrant |
| **Systemic Therapy** | n/a | n/a | platinum doublet^1^ | n/a | platinum doublet^1^ | platinum doublet^1^ | n/a | n/a^2^ | platinum doublet, liposomal doxorubicin^3^ | platinum doublet^5^ |
| **Radiation** | n/a | n/a | n/a | n/a | n/a | n/a | n/a | n/a | EBRT/VBT^4^ | n/a |

^1^ Carboplatin and paclitaxel, given in the neoadjuvant setting for presumed ovarian cancer at initial clinical consultation. PDX was created from tumor collected during interval cytoreduction.

^2^ Patient was diagnosed with stage IIIA estrogen receptor positive breast cancer 5 years prior to endometrial cancer diagnosis and underwent left mastectomy, undefined chemotherapy, and radiation followed by tamoxifen until 9 months prior to endometrial cancer diagnosis. No chemotherapy for endometrial cancer was given prior to tumor collection.

^3^ Adjuvant carboplatin and paclitaxel was given at initial diagnosis. First recurrence 1 year later was treated with liposomal doxorubicin for 5 cycles. Subsequently, after only four months of observation, disease progression was apparent on CT scans and the PDX was created from a biopsy specimen.

^4^ Adjuvant external beam radiation therapy (EBRT) and vaginal brachytherapy (VBT) was given as part of the initial management.

^5^ Neoadjuvant carboplatin and paclitaxel was given at initial diagnosis, but the disease was refractory after 6 cycles, based on CT scans and a new malignant bowel obstruction. The obstruction was managed surgically and the PDX was created from surgical specimens.

**Supplemental Table 2.** Combination index calculations for titrating concentrations of rucaparib and SN-38 at 0.5 and 0.75 fraction affected (FA). Values <1 are consistent with synergy. Genomic instability score (GIS) is included for each endometrial patient derived xenograft.

| Fa | PH412  GIS 34 | PH456  GIS 54 | PH537  GIS 81 | PH658  GIS 32 | PH750  GIS 60 | PH798  GIS 36 | UT002  GIS 29 | UT015  GIS 45 | U1561.011  GIS 47 | U1561.019  GIS 28 |
| --- | --- | --- | --- | --- | --- | --- | --- | --- | --- | --- |
| 0.50 | 0.71 | 1.08 | 0.64 | 0.83 | 0.67 | 0.63 | 0.67 | 0.75 | 0.81 | 1.4 |
| 0.75 | 0.60 | 1.25 | 0.72 | 1.32 | 0.68 | 0.70 | 0.71 | 0.88 | 0.55 | 1.2 |

**Supplemental Table 3.** Linear mixed effects models were used to compare tumor growth trajectories between treatment groups. P-values are shown for each comparison of specific groups in each PDX for the corresponding Figure (Fig). Combination = PLX038A + Rucaparib. Significance was set at p < 0.05.

| **Figure** | **PDX Model** | **Combination  vs  Control** | **PLX038A  vs  Control** | **Rucaparib  vs Control** | **Combination vs  Rucaparib** | **PLX038A  vs  Rucaparib** | **Combination vs PLX038A** |
| --- | --- | --- | --- | --- | --- | --- | --- |
| Fig. 7A | PH537 | *<0.0001* | 0.0164 | *<0.0001* | *<0.0001* | 0.1177 | *<0.0001* |
| Fig. 7B | PH658 | *<0.0001* | *<0.0001* | 0.0365 | *<0.0001* | *<0.0001* | *<0.0001* |
| Fig. 7C | PH456 | *<0.0001* | *<0.0001* | 0.1470 | <0.0001 | *<0.0001* | 0.6585 |
| Fig. 7D | U1561.019 | 0.0019 | 0.0179 | 0.4972 | 0.0006 | 0.0209 | 0.4370 |
| Fig. 7E | UT002 | *<0.0001* | *<0.0001* | 0.9648 | *<.0001* | *<0.0001* | *<0.0001* |

**Supplemental Table 4. Clinical characteristics associated with primary patient tumors.** Serous, endometrioid grade 3, and carcinosarcoma cases are listed with mismatch repair (MMR) gene expression by immunohistochemistry (IHC) of MLH1, MSH2, MSH6, and PMS2. Loss of any MMR gene expression indicates deficiency. *TP53* gene status was determined by either next generation sequencing (NGS) or IHC staining pattern. A pathogenic mutation by NGS or abnormal staining pattern by IHC was considered “aberrant”. Staging is based on FIGO 2009. Lymphovascular space invasion (LVSI) is reported at present (+) or absent (-). *UT021 was initially diagnosed with serous endometrial cancer based on a limited D&C specimen but the specimen collected for this study at the time of surgical resection revealed only endometrioid histology, grade 2.

| **Patient ID** | **Histology** | **Stage** | **LVSI** | **MMR Status** | **TP35**  **(NGS or IHC)** |
| --- | --- | --- | --- | --- | --- |
| GY008 | Serous | IVB | + | proficient | aberrant |
| UT016 | Serous | IA | - | proficient | normal |
| UT017 | Endometrioid | IIIC1 | + | MSH6 +/- | aberrant |
| UT018 | carcinosarcoma | IA | - | proficient | aberrant |
| UT019 | Serous | IVB | + | proficient | aberrant |
| UT020 | carcinosarcoma | IA | - | proficient | aberrant |
| UT021* | Endometrioid | IA | + | proficient | normal |
| UT022 | carcinosarcoma | IIIC1 | + | proficient | aberrant |
| UT023 | Endometrioid | IB | - | deficient | normal |
| UT024 | Endometrioid | IA | - | deficient | normal |
| UT025 | carcinosarcoma | IVB | + | proficient | aberrant |
| UT026 | Serous | IVB | + | proficient | aberrant |
| UT027 | Serous | IVB | + | proficient | aberrant |
| UT030 | Serous | IVB | + | proficient | aberrant |
| UT031 | Serous | IVB | + | proficient | aberrant |
| UT033 | Serous | IA | - | proficient | aberrant |
| UT034 | Serous | IIIC1 | + | deficient | aberrant |
| UT035 | Serous | IA | + | proficient | aberrant |
| UT036 | Serous | IIIC1 | + | proficient | aberrant |
| UT038 | Serous | IIIA | + | proficient | aberrant |
